# High molecular weight hyaluronic acid drastically reduces chemotherapy-induced mucositis and apoptotic cell death

**DOI:** 10.1038/s41419-023-05934-6

**Published:** 2023-07-21

**Authors:** Ali I. Mohammed, Antonio Celentano, Rita Paolini, Jun T. Low, John Silke, Lorraine A. O’ Reilly, Michael McCullough, Nicola Cirillo

**Affiliations:** 1 Melbourne Dental School, The University of Melbourne, 720 Swanston Street, 3053 Carlton, VIC Australia; 2 College of Dentistry, The University of Tikrit, Tikrit, Iraq; 3 The Walter and Eliza Hall Institute of Medical Research, 1G Royal Parade, Parkville, VIC 3052 Australia; 4 Department of Medical Biology, University of Melbourne, Parkville, VIC 3010 Australia

**Keywords:** Chemotherapy, Diarrhoea

## Abstract

Oral and intestinal mucositis (OIM) are debilitating inflammatory diseases initiated by oxidative stress, resulting in epithelial cell death and are frequently observed in cancer patients undergoing chemo-radiotherapy. There are currently few preventative strategies for this debilitating condition. Therefore, the development of a safe and effective mucositis mitigating strategy is an unmet medical need. Hyaluronic acid (HA) preparations have been tentatively used in oral mucositis. However, the protective effects of HA in chemotherapy-induced mucositis and their underlying mechanisms remain to be fully elucidated. This study aimed to assess these mechanisms using multiple formulations of enriched HA (Mucosamin^®^), cross-linked (xl-), and non-crosslinked high molecular weight HA (H-MW-HA) in an oxidative stress-induced model of human oral mucosal injury in vitro and an in vivo murine model of 5-flurouracil (5-FU)-induced oral/intestinal mucositis. All tested HA formulations protected against oxidative stress-induced damage in vitro without inducing cytotoxicity, with H-MW-HA also significantly reducing ROS production. Daily supplementation with H-MW-HA in vivo drastically reduced the severity of 5-FU-induced OIM, prevented apoptotic damage and reduced COX-2 enzyme activity in both the oral and intestinal epithelium. In 5-FU-injected mice, HA supplementation also significantly reduced serum levels of IL-6 and the chemokine CXCL1/KC, while the serum antioxidant activity of superoxide dismutase was elevated. Our data suggest that H-MW-HA attenuates 5-FU-induced OIM, at least partly, by impeding apoptosis, inhibiting of oxidative stress and suppressing inflammatory cytokines. This study supports the development of H-MW-HA preparations for preventing OIM in patients receiving chemotherapy.

## Introduction

Mucositis is a common and debilitating complication associated with the cytotoxicity of radiotherapy, chemotherapy or chemoradiotherapy (CRT) [1–3]. The condition affects the entire alimentary canal from the mouth to the anus [2, 3]. In particular, mucositis results in ulceration and pain in both the mouth and intestine that may require opioid use. This sequela can adversely affect patient’s quality of life to an extent that a delay or even cessation of cancer treatment is necessitated. Mucositis is also associated with anorexia, vomiting, and diarrhea [4–6] and remains a significant burden for up to 80% [7, 8] of those patients undergoing chemotherapeutic or radiation regimens [9–12]. Pertinently, 5-fluorouracil (5-FU) [13, 14], one of the most widely used chemotherapeutic agents for malignancies such as breast and gastrointestinal tract cancers, is commonly associated with mucositis [14].

The consequences of mucositis are far-reaching and include changes in nutritional status, gastrostomy or parenteral feeding dependence, hospitalization, and the predisposition to bacteraemia or even sepsis in severe cases. From a healthcare perspective, economic evaluations indicate considerable incremental expenditure for mild/moderate mucositis (US$1936.06, 2012 values) and severe mucositis (US$4099.89, 2012 values). This is compounded by the fact that ~400,000 patients per year in the USA alone suffer from cancer therapy induced oral mucositis (OM) [15], with an estimated value in excess of US$1 Billion/year. Hence, effective prevention of mucositis would not only improve quality of life and enable patients to receive higher therapeutic dosage of chemoradiotherapy for durable remission or even cure, but also provide substantial economic benefit [16, 17].

Despite the significant clinical and economic impact of mucositis, coagents used in its management (anti-inflammatories, biologic response modifiers, cytoprotectants antimicrobials, antifungal) are generally only palliative [12, 18–20] with only a few agents (palifermin and benzydamine) approved to date [2]. Palifermin is expensive, with use limited to patients with hematological malignancies [21], while benzydamine (approved in Europe) has limited overall activity [18, 22]. Other therapies, including local application of cryotherapy, photobiomodulation (PBM) therapy, PTA (polymyxin E, tobramycin and amphotericin B) and granulocyte macrophage-colony-stimulating factor/granulocyte colony-stimulating factor (GM-CSF/GCSF) [23] remain to be clinically validated [21]. Currently, no single intervention that universally mitigates oral or intestinal mucositis exists to both improve quality of life for cancer patients and reduce the burden on healthcare.

An understanding of the pathophysiology of chemotherapy-induced mucositis is instrumental for the development of novel mechanism-based drugs for this condition. In general, oral mucositis (erythema and ulcerative lesions) and intestinal mucositis (villi shortening and disruption crypt cell homeostasis) are thought to be due to excessive inflammation, epithelial atrophy and apoptosis, coupled with cellular hypo-proliferation, resulting in mucosal thickness reduction [24, 25]. At the molecular level, oral and intestinal chemotherapy-induced mucosal injury is associated with the production of reactive oxygen species (ROS) at early preclinical stages. Consequently, activation of oxidative stress pathways [26, 27] and DNA damage leads to basal and suprabasal epithelial cell death and subsequent breakdown of the oral or intestinal barrier [2, 3, 7, 16]. Given the vital role played by ROS in mucositis pathogenesis, inhibition of this pathway could be a valid therapeutic strategy. In this regard, anecdotal evidence suggests that hyaluronic acid (HA) may be effective in preventing oxidative stress-induced mucosal damage in vitro and it may have clinical therapeutic benefits in vivo [28]. HA is an essential constituent of connective tissues, and the most important glycosaminoglycan produced by fibroblasts during wound healing [29]. Importantly, HA is naturally biocompatible, biodegradable, non-immunogenic, and the salt form (sodium hyaluronate, SH), is currently widely used in clinical practice to accelerate skin wound repair [30]. Treatment for oral mucositis with HA has been proposed [31] and pertinently a gel based on sodium hyaluronate (SH) has received ethical approval by the FDA as a Class 1 medical device for OM-associated pain [32]. Several other HA-based compounds have also been reported to be useful in accelerated healing and pain management in clinical trials [19, 31, 33], however definitive evidence for the efficacy of HA in preventing alimentary tract mucositis is still lacking. Furthermore, the mechanisms underlying HA’s ability to prevent, mitigate, or treat mucositis are yet to be fully elucidated [28, 31, 34].

In the present study, we used a pre-clinical murine model of 5-FU-induced oral and intestinal mucositis (OIM) to investigate the clinical, histological, and molecular effects of orally administered high molecular weight hyaluronic acid (H-MW-HA) in the prevention of chemotherapy-induced mucositis in mice. Our findings have clinical ramifications and support the development of H-MW-HA preparations for preventing OIM in patients.

## Results

### HA constituents protect human oral keratinocytes against H_2_O_2_-induced cytotoxicity

Exogenous treatment of the immortalized normal human oral keratinocyte cell line (OKF6) [35], with H_2_O_2_ inhibited cell proliferation. IC_50_ was reached after 24 h of exposure to 400 μM of H_2_O_2_ (Supplementary Fig. S1). Conversely, none of the HA constituents tested (Mucosamin^®^, H-MW-HA, xl-HA 5/5, xl-HA 30/30, and xl-HA 100/100) exerted cytotoxic effects on OKF6 cells. Apart from Mucosamin^®^, all other HA constituents promoted OKF6 cell proliferation compared to the control group (Supplementary Fig. S2A–C). All HA constituents significantly counterbalanced the oxidative stress-induced cytotoxicity in OKF6 cells at all tested concentrations (Supplementary Fig. S2A, Supplementary Table S1). When tested at varying concentrations, both Mucosamin^®^ (1% and 5% v/v) and H-MW-HA (0.01% and 0.05% w/v) significantly decreased H_2_O_2_-induced cytotoxicity in OKF6 cells (Supplementary Fig. S2B and C), with the most marked protective effects observed with H-MW-HA treatment. H-MW-HA constituent (0.01% w/v, and 0.05% w/v) was therefore chosen for further experiments investigating H_2_O_2_-mediated ROS production in immortalized normal human oral keratinocytes.

### H-MW-HA protects human oral keratinocytes against H_2_O_2_-induced ROS production

We next monitored changes in the intracellular redox state by introducing a fluorescent dye probe in OKF6 cells, based on the redox-active green fluorescent (5-(and-6)-chloromethyl-2',7'-dichlorodihydrofluorescein diacetate (CM-H_2_DCFDA). Upon exposure to H-MW-HA either in the presence or absence of H_2_O_2_-induced oxidative stress, ROS production was monitored over a 6-h period. While H-MW-HA (0.01% w/v and 0.05% w/v) did not exert any significant effect (Supplementary Fig. S3), the addition of H_2_O_2_ resulted in a rapid increase in ROS levels in all experimental groups (Supplementary Fig. S3). A significant reduction in ROS levels in the H-MW-HA (0.05% w/v) + H_2_O_2_ (400 μM) group compared to the H_2_O_2_ (400 μM) treated group commenced as early as 30 min and continued during over the 6-hour monitoring period (Supplementary Fig. S3). Our data also showed that ROS production in the H_2_O_2_ treated group plateaued at two time points (0.5–1 and 3–4.5 h post treatment; Supplementary Fig. S3). H-MW-HA at both concentrations (0.01% w/v and 0.05% w/v) significantly reduced (*p* < 0.001) ROS levels of production (Supplementary Table S2). Therefore, the reduction in the H_2_O_2_-induced ROS production levels in OKF6 cells treated with 0.01% w/v and 0.05% w/v H-MW-HA could be attributed to the protective effect of the HA.

### H-MW-HA improves survival of 5-FU treated mice

**Fig. 1:**
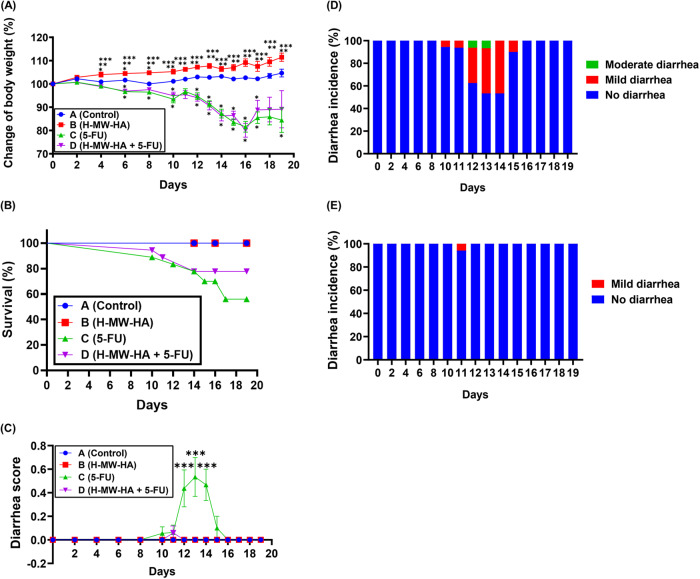
The effects of high molecular weight hyaluronic acid (H-MW-HA) on body weight rate, survival, and diarrhea induced by 5-flurouracil (5-FU) in mice. **A** Changes in body weight observed in mice over time during HA administration while on 5-FU; Body weight of mice was measured every 24–48 h. The percentage of body weight of every mouse was calculated and compared with the percent of body weight at day 0. The mean body weight of every group (A, B, C, and D) at day-0 was defined as 100%. Mean ± SEM are showed. *p < .05 vs control group; **p < 0.05 vs 5-FU; ***p < 0.05 vs 5-FU + H-MW-HA. **B** Time-course of survival during 5-FU treatment. Mean ± SEM are showed. **C** Diarrhea score. Score: 0, no diarrhea; 1, mild diarrhea; 2, moderate diarrhea; 3, severe diarrhea. The data shown are means ± SEM. **D** Incidence (%) and severity of diarrhea following 5-FU treatment. **E** Incidence (%) and severity of diarrhea following H-MW-HA administration in 5-FU treated mice. Administration of H-MW-HA in drinking water significantly ameliorated chemotherapy‑induced diarrhea. The occurrence of severe and moderate diarrhea was prevented in mice treated with H-MW-HA before, during, and after 5-FU regimen. ***p < 0.001 versus control, H-MW-HA, and 5-FU + H-MW-HA group. (*n* = 12, 12, 18, and 18 for groups A, B, C, and D, respectively). Group A; Saline (normal control); Group B; H-MW-HA (treatment only); Group C; 5-FU (positive control); Group D; 5-FU + H-MW-HA.

Subsequently, H-MW-HA was tested in our dual murine model of OIM. Mice were divided into four groups, namely “A” (control), “B” (H-MW-HA), “C” (5-FU), and “D” (H-MW-HA + 5-FU) (Supplementary Fig. S4). Maximal body weight (BW) loss in groups C and D were observed on day 16 after 5-FU induction (*p* < 0.05) (Fig. 1A). By day 19, significant differences between group C (5-FU) and groups A (control) and B (H-MW-HA) (*p* < 0.05, Supplementary Table S3) were observed. Mice treated with H-MW-HA + 5-FU (group D) also exhibited a trend of reduced body weight loss (Fig. 1A). Since weight loss in excess of 20% was deemed unethical, death/drop out was recorded as those mice with >20% weight loss [36]. Mortality (death or weight loss over 20%, Fig. 1B) in the 5-FU treated group (44%) was significantly higher (*p* = 0.03) than that of the H-MW-HA + 5-FU group (22.2%). In summary, H-MW-HA treatment of mice receiving 5-FU (group D) did not significantly improve weight loss but significantly improved overall survival, compared to those animals receiving 5-FU alone (group C).

### H-MW-HA ablates chemotherapy-induced diarrhea

Control (A) and H-HM-W only (B) treated groups exhibited no obvious diarrheal symptoms during the entire experiment period (Fig. 1C). Group C, treated with 5-FU alone developed diarrhea from day 9, peaking at day 13 of 5-FU treatment (Fig. 1C, D). Overall, diarrhea developed in 72.2% of mice in the 5-FU treated group (group C), which was dramatically reduced to 5.6% with H-MW-HA supplementation (0.01% w/v, group D, Supplementary Table S4). Group D (H-MW-HA + 5-FU) also displayed a less severe and transient pattern of diarrhea from day 11, which resolved within 24 h (Fig. 1C, E). Stool consistency score was significantly reduced (*p* < 0.001) in the 5-FU treated group also receiving H-MW-HA (group D) on days 12, 13, and 14, compared to 5-FU alone, to levels similar to those of the control (A) and H-HM-W (B) treated groups (Fig. 1C). A chi-square test of independence was performed to examine the correlation between the treatments (5-FU, and H-MW-HA) and the development of diarrhea (present/absent) in our treated groups. We found a significant association between the presence of diarrhea and 5-FU treatment (chi-square test; χ^2^ (1) = 16.831, *p* = 0.00004, Cramer’s *V* = 0.684, implying a relationship between diarrhea status and 5-FU treatment. In summary, mice from the 5-FU group (C) had a higher risk of developing diarrhea compared to control group (A), while H-MW-HA treatment significantly reduced the occurrence of 5-FU-induced diarrhea.

### H-MW-HA significantly reduces intestinal damage in 5-FU treated mice

**Fig. 2:**
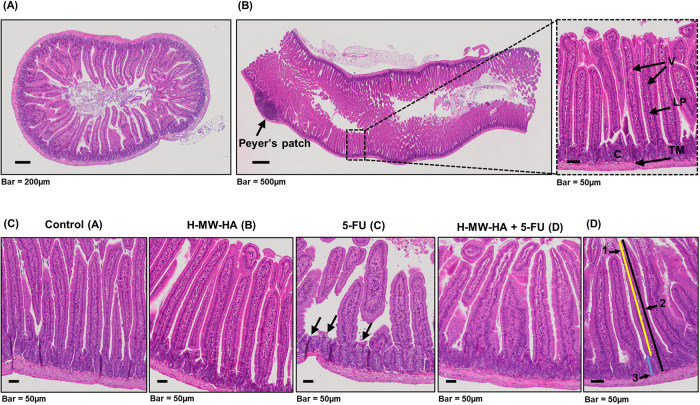
Effect of H-MW-HA on the 5-FU-induced intestinal mucosa damage. Histological changes in the intestinal villi were determined using H&E staining. **A** Representative microphotograph showing the histologic section of a transversely cut intestinal segment of the control animal. **B** Representative microphotograph showing the histologic section of a longitudinally cut intestinal segment of a control animal. The tunica propria forms the core of the villi and consists of connective tissue, elastic and reticular fibers and lymphoid cells, below which extends the muscular mucosa consisting of smooth muscle cells. Beneath the mucosa lies the tunica submucosa, a layer of loose to dense connective tissue containing blood and lymphatic vessels. Tunica muscularis is the outer covering layer of the intestine and consists of two thick layers of smooth muscle. Structural landmarks: Villi (V), Crypts (C), lamina propria (LP), Tunica muscularis (TM). **C** Representative microphotograph showing histological appearances of the intestinal mucosa of control (**A**), H-MW-HA-treated (**B**), 5-FU-treated (**C**), and H-MW-HA + 5-FU-treated (**D**) mice. Compared with control mice, the innermost layer was largely destroyed in 5-FU treated mice (**C**), and the epithelial thickness was reduced (epithelial atrophy), with the villi length shortening and thinning of lamina propria (black arrows) with inflammatory cell infiltration. The same histological aspects were confirmed in multiple Control (**A**), H-MW-HA-treated (**B**), 5-FU-treated (**C**), and H-MW-HA + 5-FU-treated (**D**) group. **D** Intestinal thickness measurements; 1, villus length (yellow line); 2, tunica mucosa thickness (black line); 3, crypt depth (blue line).

Assessment of mucositis severity was also performed by histopathological scoring of H&E stained jejunum, (Fig. 2A, B) in 5-FU and control-treated mice. Both control and H-MW-HA only treated groups (groups A and B) showed no histopathological changes during the time-course (clear intestinal mucosa, intact epithelium, tall and columnar epithelial cells, villi aligned, crypts intact, Fig. 2C, groups A and B). In contrast, 5-FU treatment resulted in a marked reduction in the integrity and architecture of intestinal mucosa and severe pathology, including epithelial atrophy, villus length reduction, and thinning of the lamina propria with inflammatory cell infiltration (Fig. 2C, group C). Strikingly, these histopathological features were reduced with the addition of H-MW-HA to levels observed in the control groups (Fig. 2C, group D).

**Fig. 3:**
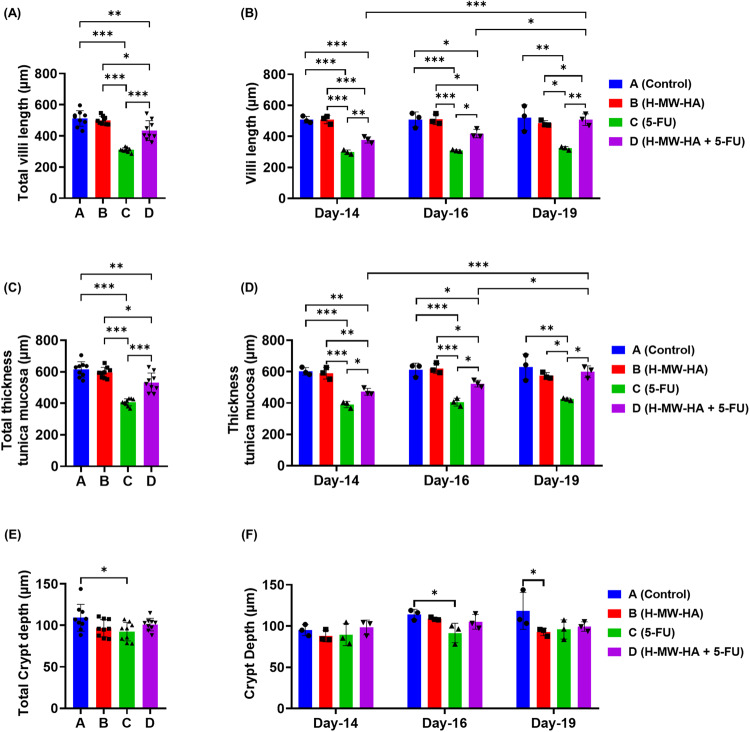
H-MW-HA reduced intestinal damage in 5-FU treated mice. **A** Total villi length (average) of the mice jejunum between Day-14 and Day-19. **B** The average villi length of the jejunum at each time point. **C** Total thickness of tunica mucosa (average) of the mice jejunum between Day-14 and Day-19 **D** The average tunica mucosa thickness of the jejunum at each timepoint. **E** Total crypt depth (average) of the mice jejunum between Day-14 and Day-19. **F** The average crypt depth of the jejunum at each timepoint. Data represented as mean ± SD. *p < 0.05. **p < 0.005. ***p < 0.001; *n* = 9 mice/group, 3 mice/group/timepoint, and 12 representative measurement/tissue.

As a further marker of intestinal integrity, we measured villus length, thickness of tunica mucosa, and crypt depth (Fig. 2D). Villus length (Fig. 3A, B) was significantly decreased in the jejunum of the 5-FU treated mice (group C, 310.35 ± 14.02 µm) compared to the control and H-MW-HA alone treated groups (groups A and B, 511.92 ± 49.73 µm, and 500.66 ± 25.93 µm; respectively, *p* < 0.0001, Fig. 3A, Supplementary Table S5). Villus length in the 5-FU + H-MW-HA treatment group (group D) was less significantly impacted compared to the 5-FU group (434.44 ± 63.11 µm; *p* < 0.0001) with abundant preservation of normal villus architecture (Fig. 3A).

Further morphometric analyses of H&E stained jejunum performed by measuring the thickness of the tunica mucosa and crypt depth revealed a significant reduction in the thickness of the intestinal tunica mucosa in the 5-FU group (C) compared to the control and H-MW-HA alone treated groups (A and B) between day 14 and 19 (Fig. 3C) and on days 14, 16 and 19 (Fig. 3D). In contrast, H-MW-HA supplementation to the 5-FU treated mice in group (D), significantly alleviated the severity of tunica mucosa thickness loss compared to the 5-FU group (C) on days 14, 16 and 19 (Fig. 3C, D). H-MW-HA treatment of 5-FU treated mice (group D) did not affect the crypt depth (Fig. 3E, F, Supplementary Table S5).

In conclusion, our jejunum morphometric analyses demonstrated that 5-FU administration instigated severe intestinal mucosal damage, which was dramatically reduced by co-treatment with H-MW-HA.

### H-MW-HA decreases severity and duration of chemotherapy-induced oral mucositis in mice

**Fig. 4:**
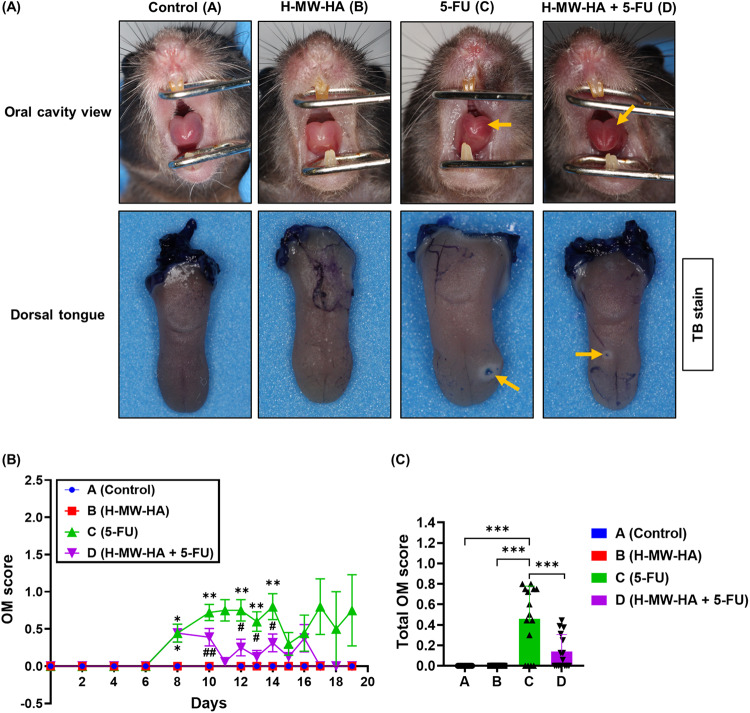
Effect of H-MW-HA on oral mucositis scores in 5-FU treated mice. Oral mucositis (OM) was scored on a six-point scale as described in methods. **A** Representative photographs represent oral mucosa of the mice showing diffuse erythema on the anterodorsal surface of the tongue in treated mice after TB staining showing the epithelium ulceration (in blue) at the dorsal surface of the tongue. Yellow arrows show areas of erythema, erosion, and ulceration. **B** Comparative mucositis scores. Data are the mean ± SEM. *p < 0.01 versus control (A) and H-MW-HA (B) groups; **p < 0.01 versus control (A), H-MW-HA (B), and H-MW-HA + 5-FU (D) groups; ^#^*p* < 0.01 versus 5-FU group; ^##^*p* < 0.01 versus control (A), H-MW-HA (B), and 5-FU (C) groups (Tukey’s post hoc test). (**C**) Mean value of the total score of oral mucositis between day-0 and day-19 for each group. Data are the mean ± SEM. *p = 0.0191; ***p < 0.0001 (Tukey’s post hoc test).

In order to assess the potential protective effects of HA against chemotherapy-induced oral mucositis (CIOM), the murine oral cavity (groups A–D; Supplementary Fig. S5A) were visually assessed using specialized tailored oral cavity diagnostic tools (Supplementary Fig. S5B and B) and an incremental scoring system (Supplementary Table S6) adopted from a previous study [37]. We found that i.v. administration of 5-FU induced development of OM in the 5-FU and H-MW-HA + 5-FU treated groups (groups C and D), whereas control and H-MW-HA groups (groups A, and B) did not develop disease (Fig. 4A–C). From day 8 of 5-FU exposure, mucositis in the 5-FU only treatment group (group C) continued to increase in severity, reaching peaks at days 14 and 17 (Fig. 4B). At termination of the study, group C had an OM score of 0.75 ± 0.47 (Fig. 4B), in contrast, mice receiving H-MW-HA + 5-FU (group D), had a significantly lower OM score on days-10, 12, 13, and 14 (Fig. 4B, C*p* < 0.01 and *p* < 0.001 respectively). From day 14, the H-MW-HA + 5-FU (group D) OM began to decrease and by day 17, the mucosa of the remaining mice was completely healed (Fig. 4B).

**Fig. 5:**
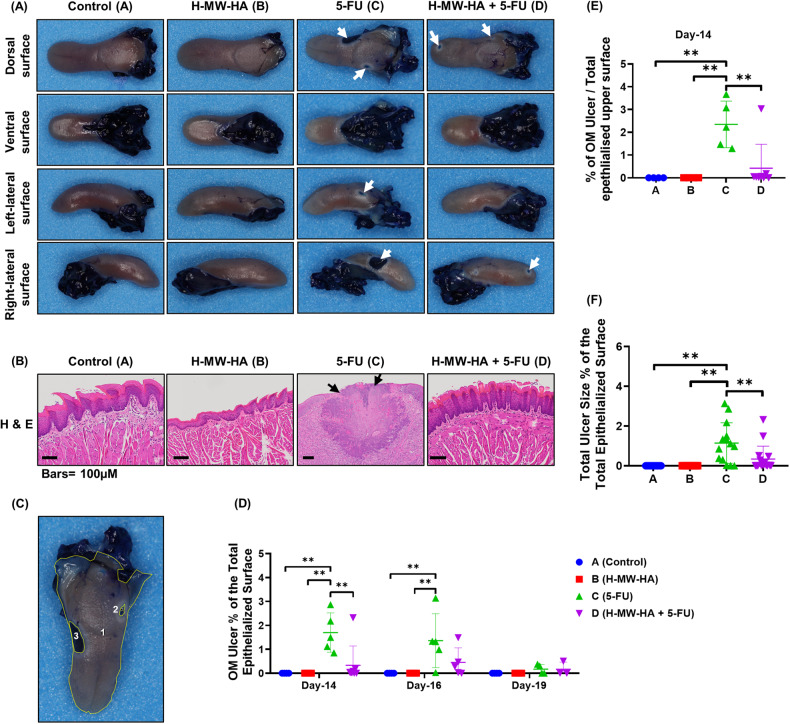
Effect of H-MW-HA on chemotherapy-induced oral mucositis (CIOM) ulcer size percentage in 5-FU-treated mice. Animals were sacrificed at different time points, and their tongues were collected for analysis. The harvested tongues were stained with 1% toluidine blue (TB) in 10% acetic acid. After imaging the tongues, the ulcer size and the total epithelialized upper surface area of the tongue were measured for all experimental groups (**A**–**D**). **A** Representative photographs showing the tongues of mice after staining with 1% toluidine blue (TB) in 10% acetic acid. The blue areas indicate CIOM ulcers at different surfaces of the tongue (white arrows). **B** Histological sections of the tongues stained with H&E in each experimental group. The black arrows indicate areas of erosion. **C** Representative photograph of a TB-stained tongue surface, highlighting the ulcerated epithelium (blue) on the upper surface of the tongue. For quantification, the ulcer size percentage of chemotherapy-induced oral mucositis (CIOM) relative to the total epithelialized surface of the tongue was measured using Fiji (ImageJ) software. The ulcer size percentage was calculated as the TB-positive surface area of the tongue (excluding excision trauma) divided by the total epithelialized surface area of the tongue. The numbered areas indicate the selected measured surface areas. **D** Mean percentage of oral mucositis (OM) ulcer size relative to the total epithelialized surface of the tongue at days 14, 16, and 19. **E** Mean percentage of OM ulcer size relative to the total epithelialized upper surface of the tongue on day 14. **F** Total mean percentage of OM ulcer size relative to the total epithelialized surface of the tongue between days 14 and 19. Data from days 14, 16, and 19 presented as the mean ± SEM. **p *<* 0.005 (*n* = 12, 12, 15, and 16 for groups **A**, **B**, **C**, and **D**, respectively).

To explore the clinical signs of mucositis more thoroughly in our murine model, we conducted the first in-depth chemotherapy-induced ulceration assessment. We found that OM ulceration was localized mainly to the tongue dorsolateral and ventral surfaces (Fig. 5A). Positive areas of oral ulcerative mucositis were revealed by toluidine blue (TB, deep blue coloration) (Fig. 5A, white arrows), which corresponded to erosive/ulcerative lesions with extremely atrophic or absent epithelium (Fig. 5B, group C). TB-stained tongue CIOM ulceration was present in 85.71% of animals in the 5-FU only treated group (C) but only 68.75% of mice also treated with H-MW-HA (group D). Importantly, a strong association between CIOM ulcer status (present/absent) and 5-FU treatment was observed; χ^2^ (3) = 32.93, *p* < 0.0001, Cramer’s *V* = 0.781. Quantitative analysis of the dorsal and ventral tongue surfaces (Fig. 5C) showed that 5-FU treated animals had a significantly larger ulcer mean size epithelial surface percentage at day-14, and day 16 (Fig. 5D). At this timepoint, the CIOM ulcer size percentage compared to the total epithelialized upper surface of the tongue was also the highest in the 5-FU only treated mice (group C, 2.34 ± 0.45 % Fig. 5E) and significantly elevated (*p* < 0.005) compared to all other treatment groups (groups A, B, and D, Fig. 5E). H-MW-HA supplementation to the 5-FU treated mice in group (D), significantly alleviated the severity of CIOM and reduced the ulcer size compared to the 5-FU treated mice (Fig. 5D, E). The total mean size percentage of CIOM ulcer, relative to the total epithelialized surface of the tongue between days 14 and 19 for animals treated with H-MW-HA + 5-FU (group D) was significantly (*p* < 0.005) lower than the ulcer percentage for those treated with 5-FU alone (group C, Fig. 5F). These results provide the first demonstration that H-MW-HA can mitigate CIOM by decreasing both disease severity and duration.

### H-MW-HA significantly reduces oral mucosal damage in the tongue of 5-FU-treated mice

**Fig. 6:**
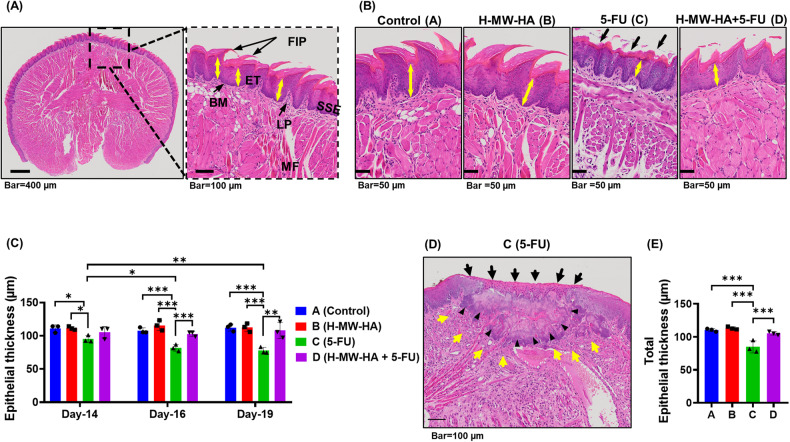
Effect of H-MW-HA on the 5-FU-induced tongue mucosa damage. Histological changes in the tongue were determined using H&E staining. **A** Representative microphotograph showing the histologic section of the base of the tongue of the control animal. Basal cells are just above the basement membrane and more superficially is the succeeding prickle, granular, and squamous layers. The basement membrane is a thin mat of the extracellular matrix that separates epithelial sheets from connective tissue. Underlying stroma is composed of connective tissue that contains muscle fibers, blood vessels, and nerves, fibroblasts and can include minor salivary glands. Structural landmarks: filiform papillae (FIP), stratified squamous keratinized epithelium (SSE), basement membrane (BM), lamina propria (LP), and muscle fibers (MF). **B** Representative microphotograph showing histological appearances of the tongue mucosa of control (group A), H-MW-HA (group B), 5-FU (group C), and 5-FU + H-MW-HA (group D)-treated mice. Compared with control mice, the superficial regions were largely destroyed in 5-FU treated mice (C), and the epithelial thickness was reduced (epithelial atrophy), with the filiform papilla being entirely damaged (black arrows), and the connective tissue showed a marked increase in infiltrating cells as a sign of the inflammatory response. **C** The average epithelial thickness of the dorsal tongue at each time point (27 representative measurement/tissue (three representative mice/group). Data represented as mean ± SD. *p < 0.05. **p < 0.005. ***p < 0.001. **D** Representative microphotograph showing ulcerative lesions (arrows head) covered by a thin layer of necrotic fibrinoid material at the base and margins (black arrows) with regions containing of inflammatory cells and active granulation tissue (yellow arrows). **E** The total epithelial thickness of the dorsal tongue between Day-14 and Day-19; each dot is the average of 27 representative measurements/tissue. Data represented as mean ± SD. *p < 0.05. **p < 0.005. ***p < 0.001.

Analysis of H&E sections on days 14, 16, and 19 showed that between days 14 and 19, the normal tongue pathology (Fig. 6A) in both control (group A) and H-MW-HA (group B) treated groups was preserved with no visible lesions (Fig. 6B, groups A, and B). In contrast 5-FU administration caused epithelial atrophy (Fig. 6B, groups C, and D), reflected by a significant reduction (*p* < 0.001) in epithelial thickness (yellow double-headed arrows) compared to the control groups (groups A and B, Fig. 6C). In addition, 5-FU treatment also affected the structural integrity of the dorsal mucosa, resulting in complete destruction of the filiform papillae, a change in appearance of the uniform keratinized layer to a desquamative appearance (Fig. 6B, group C), and severe changes consistent with ulceration of the tongue mucosa (Fig. 6D). Importantly, H-MW-HA supplementation protected the tongue epithelium from 5-FU-induced damage (Fig. 6B, group D). Strikingly, epithelia integrity was preserved to the degree observed in the control groups (groups A, and B, Fig. 6C, E) with filiform papillae also more frequently intact (Fig. 6B, group D). Accordingly, marked differences were found in the total epithelial thickness of the tongue mucosa between day-14 and day-19 for mice exposed to 5-FU compared to those co-treated with H-MW-HA (group D, Fig. 6C, E). In conclusion, 5-FU induced severe histological tongue damage which was normalised by co-treatment with H-MW-HA.

### H-MW-HA reduces 5-FU-induced apoptosis in the oral and intestinal mucosa

**Fig. 7:**
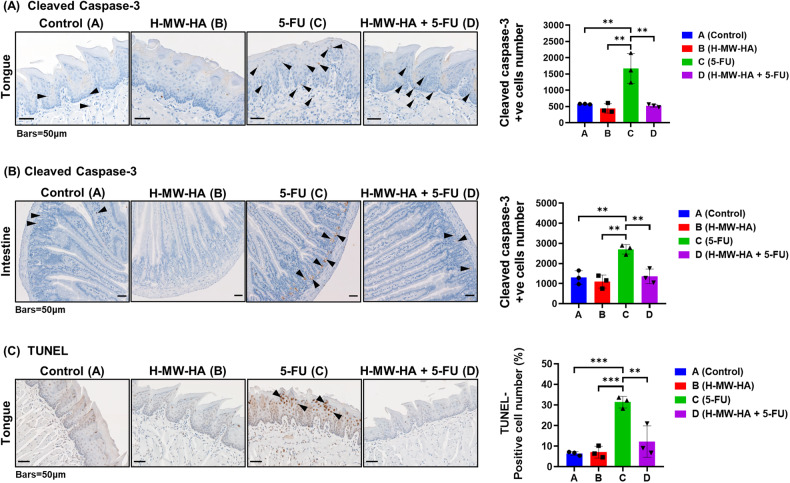
Effect of H-MW-HA on apoptosis and cleaved caspase-3 activation in the oral and intestinal mucosa induced by 5-fluorouracil (5-FU). 5-FU was injected i.v. while H-MW-HA treatment (0.01%w/v) was daily administered orally in the drinking water as described in the method. Mice were sacrificed at day 16. The tongue and jejunum were excised, and sectioned. **A** Cleaved-caspases-3 immunostaining of tongue, **B** cleaved-caspases-3 immunostaining of intestinal tissue, **C** TUNEL stain of tongue tissue were performed (images at magnification 20X; scale bar represents 50 μm and apply to all panels). Black arrow heads point to the positive cell staining. The number of apoptotic cells in tongue mucosa (A, three different transverse sections/tongue mucosa, three representative mice/group), caspase-3-activated cells in tongue mucosa (B, six random fields of tongue mucosa/tongue section, three different transverse sections/tongue, three representative mice/group), and caspase-3-activated cells in intestinal mucosa (C, twelve full villus/section, four sections/mice intestine, three representative mice/group) were counted. Data are presented as the means ± SD. where, **p < 0.005, and ***p < 0.001.

At a molecular level, mucositis initiation induces DNA strands breaks leading to the activation of executioner caspases and mucosal atrophy associated with apoptotic changes [38]. Therefore, we assessed activated caspase-3 activity on day 16. Administration of 5-FU resulted in a more than twofold increase (*p* < 0.005) in the number of cleaved caspase-3 positive cells in tongue mucosa and intestinal crypts on day 16 (Fig. 7A, B, arrow heads) compared to control animals. Administration of H-MW-HA (0.01% w/v) to 5-FU treated mice (group D) significantly suppressed the activation of caspase-3 in both tongue mucosa and intestinal crypt to the levels of control animals (Fig. 7A, B, inhibition reduction of 49.8% and 68.61% for tongue mucosa and intestine, respectively). Consistent with the significant reduction in tongue mucosal epithelial thickness (Fig. 6C, E), epithelial cell apoptosis was also significantly elevated with 5-FU administration, as demonstrated by a marked elevation in cleaved caspase-3- and TUNEL-positive apoptotic cells in tongue mucosa on day 16 (Fig. 7A, C, arrow heads). Once again, daily administration of H-MW-HA (0.01% w/v) to 5-FU treated mice (group D) significantly reduced (*p* < 0.005) the incidence of apoptotic cells to a level similar to that of the control groups (groups A, B) (Fig. 7C).

### H-MW-HA supplementation decreases 5-FU-induced inflammatory mediators and increases anti-oxidant defense

**Fig. 8:**
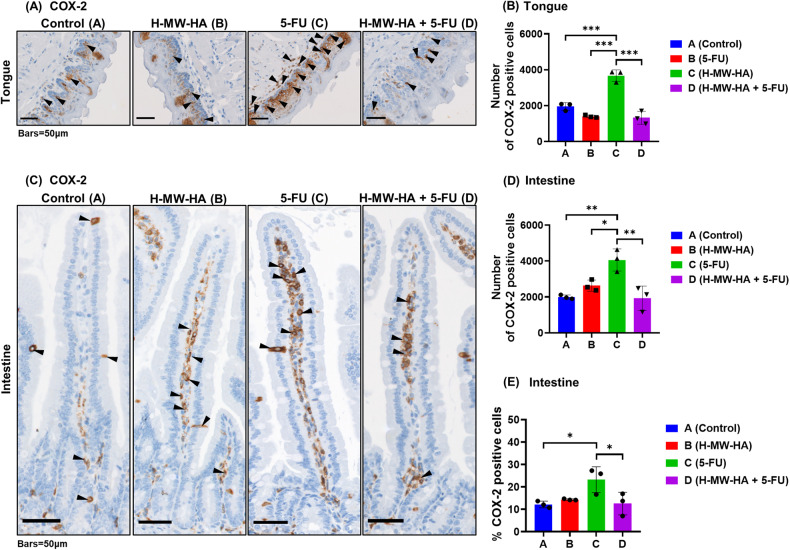
The effect of H-MW-HA on COX-2 expression in mice oral and intestinal mucosa. Immunohistochemical analysis for COX-2 expression. Mice received 5-FU intravenously (IV) with or without daily treatment of H-MW-HA (0.01%w/v) in the drinking water as described in the method. Mice were sacrificed at day 16. The jejunum were excised, and sectioned. COX-2 immunostaining of tongue and intestinal tissue were performed and COX-2 immunolabelled cells in tongue and intestinal mucosa were counted. For tongue mucosa: six random fields of tongue mucosa/tongue section, three different transverse sections/tongue, three representative mice/group. For intestine: twelve full villus per section, four intestinal sections per mice, three representative mice per group. **A** Representative microphotograph showing immunohistochemistry staining for COX-2 in Tongue mucosa of C57BL/6 mice (images at magnification X20). COX-2 positive cells were detected in the epithelial and lamina propria area (black arrowheads; bar indicates 50 μm). **B** A number of cells immunolabelled for COX-2. **C** Representative microphotograph showing immunohistochemistry staining for COX-2 in small intestine of C57BL/6 mice (images at magnification X20). COX-2 positive cells were detected in the epithelial and lamina propria area (black arrowheads; bar indicates 50 μm). **D** A number of cells immunolabelled for COX-2. **E** % of immunolabeled cells for COX-2. Data are expressed as mean ± SD. Where *p < 0.05, **p < 0.005, and ***p < 0.001.

Aligned with the oral mucosal erythema, erosions, and ulcerations in 5-FU treated mice (group C), the present study demonstrates that expression of Cyclooxygenase-2 (COX-2), a key inducible enzyme upregulated by inflammatory stimuli, was upregulated in the oral and intestinal mucosa of mice treated with 5-FU (Fig. 8). Specifically, within the tongue mucosa of 5-FU treated mice (group C), immunoreactivity of COX-2 enzyme increased significantly (*p* < 0.001) when compared to control groups (A and B; Fig. 8A, B). Moreover, the observed elevation in COX-2 immunoreactivity induced by 5-FU on the oral mucosa was reversed (*p* < 0.001) with H-MW-HA 0.01% w/v, (Fig.8A, B). Similarly, in the jejunum, the 5-FU treated group (C) also showed significantly increased COX-2 immunoreactivity (23.26 ± 5.78%, *p* < 0.05), compared to control animals (12.16 ± 1.48%, and 14.35 ± 0.33 % in groups A and B, respectively). Treatment with 0.01 % w/v H-MW-HA significantly decreased COX-2 immunoreactivity (*p* < 0.05, compared to the 5-FU treated group, D) to a similar level of control treated groups (A and B) (12.57 ± 5.06%, (Fig. 8D, E).

**Fig. 9:**
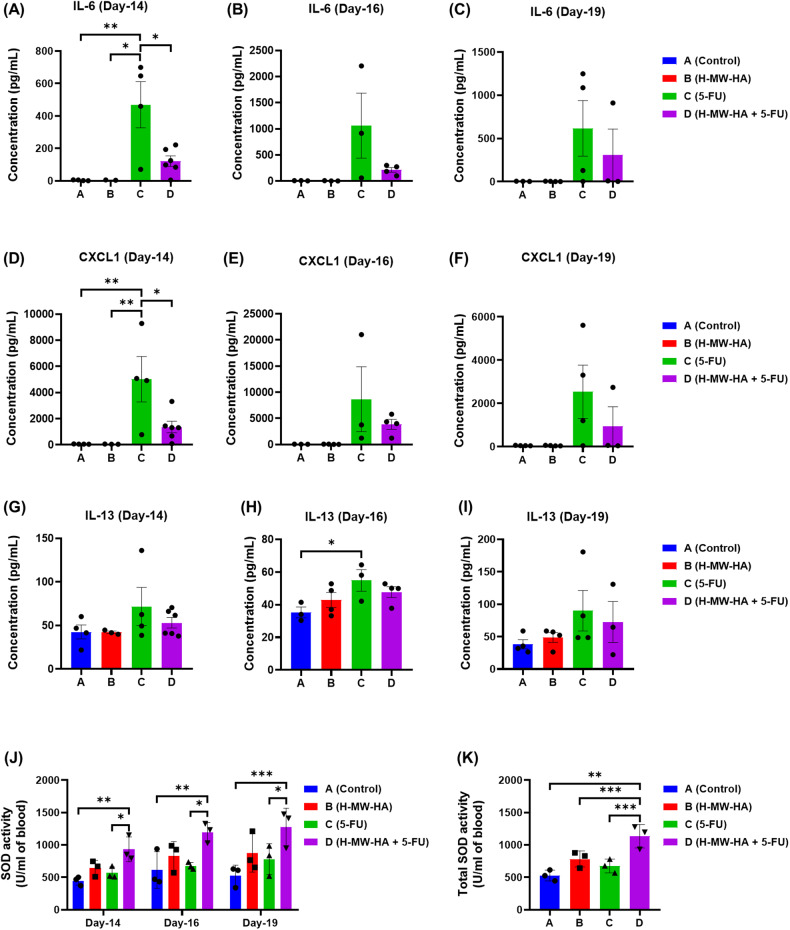
Effect of H-MW-HA on increased IL-6, KC, and IL-13 expression induced by 5-fluorouracil (5-FU), and on superoxide dismutase (SOD) enzyme activity in 5-FU treated mice. Mice received 5-FU Intravenously (IV) with or without daily treatment of H-MW-HA (0.01%w/v) in the drinking water as described in the text. Animals were sacrificed at days 14, 16, and 19, and Blood samples were obtained. The expression of pro-Inflammatory cytokine protein levels, **A**–**C** IL-6, **D**–**F** CXCL1/KC, and **G**–**I** IL-13, was determined in serum at days 14, 16, and 19. SOD activity was measured in serum. **J** SOD activity at days 14, 16, and 19. **K** Total SOD activity between day-14 and day-19. Data are expressed as mean ± SD. *p < 0.05. **p < 0.005. ***p < 0.0001.

In addition to changes in defined organs, we also found systemic change associated with the 5-FU treatment (group C), reflected as an elevation in serum expression of pro-inflammatory mediators, including IL-6 and CXCL1/KC. Compared to control and H-MW-HA treated mice (group A and B) the 5-FU (Group C) only treated mice had elevated levels of IL-6 and CXCL1/KC (Fig. 9A–F). Pertinently, elevated levels of the cytokine IL-6 and the chemokine CXCL1 are also associated with oral mucositis in humans [11, 37, 39]. In particular, day 14 of i.v. 5-FU treatment was associated with a significant increase in serum levels of IL-6 and CXCL1/KC (Fig. 9A, D), which remained elevated until experimental endpoint (day 19, Fig. 9A–F). Interestingly, higher serum levels of the anti-inflammatory cytokine IL-13, which linked to significantly elevated levels in intestinal mucositis in mice [40], was also observed on day 16 in 5-FU treated mice (group C, Fig. 9G–I). Administration of H-MW-HA (0.01% w/v) significantly attenuated the upregulation of both IL-6 and CXCL1/KC serum levels induced by 5-FU (group D), but not IL-13, indicating that the H-MW-HA exerted an anti-inflammatory effect (Fig. 9A–I). In addition, serum SOD enzyme activity, a key anti-oxidant [41], was measured at days 14, 16, and 19 (Fig. 9J). Control group mice (group A) exhibited a low basal level of serum SOD activity, which was not significantly different from either the H-MW-HA (group B) or 5-FU alone (group C) treated mice. However, mice treated with H-MW-HA + 5-FU (group D) showed significantly higher levels of serum SOD enzyme activity on all days tested compared to the control and 5-FU treated mice (group A and D) (*p* < 0.05 and *p* < 0.005, respectively; Fig. 9J). Total serum SOD activity between days 14 and 19 for animals treated with 5-FU + H-MW-HA (group D) was also significantly higher compared with those in control (group A), H-MW-HA only (group B), or 5-FU only (group C) groups (Fig. 9K).

Our data indicate that H-MW-HA 0.01% w/v exhibited anti-apoptotic, anti-inflammatory and antioxidant effects against 5-FU-induced oxidative damage and provide a possible mechanism for the H-MW-HA mediated reduction in OIM.

## Discussion

The present study has clearly demonstrated that H-MW-HA treatment dramatically reduced the clinical, histological and molecular effects of 5-FU in the oral and intestinal mucosa. In particular, H-MW-HA supplementation was associated with anti-apoptotic, antioxidant and anti-inflammatory effects in a pre-clinical model of 5-FU-induced OIM.

Oral and gastrointestinal mucositis are debilitating adverse effects observed in nearly all cancer patients undergoing chemotherapy [2, 3, 42–46]. Importantly, since they also share common underlying mechanisms, similar treatment strategies may be applicable. In the present study i.v. administration of 5-FU in mice resulted in ulcerative oral mucositis, moderate diarrhea, bodyweight loss, and damaged intestinal mucosa, mimicking the clinical scenario observed in patients, particularly, after chemotherapy treatment regimens [25, 47]. With these considerations in mind, our pre-clinical animal model of chemotherapy-induced oral and intestinal mucositis (CIOIM) [36] represents a significant improvement over previous mucositis models [37, 48] and was suitable for assessing the efficacy of a novel intervention for the prevention of chemotherapy-induced mucosal injury of the alimentary tract in vivo.

In the present study, 5-FU-induced mucosal damage in C57BL/6 mice was significantly ameliorated or abrogated by H-MW-HA co-treatment at both clinical, histological and molecular levels. Clinically, daily administration of H-MW-HA delayed the onset and ameliorated the duration and severity of 5-FU-induced diarrhea. In addition, animals receiving H-MW-HA showed improved survival rates compared to mice receiving 5-FU alone. However, the survival rate of mice beyond 14 days was low when treated with i.v. 5-FU (50 mg/kg/day). This was likely be due to severe intestinal mucositis resulting in irreversible weight loss and mice dehydration [49]. Moreover, a significant improvement in CIOIM animal survival was evident when mice were additionally treated with H-MW-HA.

In terms of oral disease, i.v. administration of 5-FU triggered a diffuse erythema and/or ulceration on the dorsal and ventral surface of the tongue. H-MW-HA significantly limited OM in the 5-FU treated mice and reduced the CIOM ulcer size, resulting in a dramatic improvement in OM. Ultimately, significantly fewer 5-FU treated mice co-treated with H-MW-HA developed oral mucosal ulcers in addition to a significant decrease in mucositis scores.

Histologically, 5-FU treated mice showed a reduction in epithelial thickness and a distortion in the structural integrity of the dorsal tongue mucosa, with filiform papillae completely effaced and the keratinized layer reduced or absent. Intestinal histological damage was also observed after 5-FU administration, resulting in major histological damage; shortening of villi length, distortion of crypts, excessive inflammatory cell infiltration and intestinal mucosa cellular damage. Typically, following the generation of ROS, the activation of NF-κB results in an upregulation of proinflammatory cytokines, including IL-6 as well as COX2. Our findings are consistent with these observations and with previous human studies [2, 3, 9, 45] in which epithelial damage and inflammatory cell infiltration in the mucosa during the inflammatory stage of mucositis have been reported. In the murine model of 5-FU-induced oral mucositis, Bertolini et al. [37] observed macroscopic/microscopic lesions and elevated proinflammatory cytokine activity such as, NF-κB, IL-1B, TNFα, CXCL1/KC, GM-CSF, and IL-6, with neutrophil infiltration. Other pre-clinical studies have also shown intestinal villous atrophy post 5-FU treatment [50]. In a mouse model of intestinal mucositis [51], intestinal injury was accompanied by decreased epithelial cell proliferation, resulting in a blunting of villus height, crypt distortion with increased crypt cell apoptosis and abundant inflammatory cell infiltration [51, 52]. This in turn resulting in impaired function, which were also observed in the present study. Importantly, our data demonstrates that these types of injury to the tongue and intestine induced by 5-FU were ameliorated by co-administration of H-MW-HA. Notably, tongue epithelial thickness and jejunum villi length recovered spontaneously and without an inflammatory cell infiltrate. Importantly, the results of this study suggest novel protective effects of H-MW-HA for attenuation of 5-FU-induced OIM. These data are invaluable for future assessment of the influence of H-MW-HA in 5-FU tumor models and ultimately of clinical relevance. However, treatment with H-MW-HA would be unjustified if it caused the growth of tumor cells or inhibited the antineoplastic effects of CT. This concern was diminished in a mouse model of ovarian carcinogenesis. Instead, SN-38, the active metabolite of irinotecan (CPT 11), conjugation to HA significantly improved the profile of in vivo tolerability and enhanced therapeutic efficacy for ovarian cancer treatment, demonstrating tumor suppressor property as was observed by Montagner et al. [53]. Further studies have also reported that HA derivatives and different types of HA-paclitaxel conjugates have potential synergistic antitumor effects [54–58]. Furthermore, combinations of antioxidants have been shown to have greater potential synergistic anticancer effects in vivo [59]. However, whether this mechanism is occurring in the combination treatment of 5-FU and H-MW-HA needs further investigation.

Molecularly, it is well-established that oxidative stress, inflammation, and apoptosis are major contributors to the development of mucositis induced by 5-FU treatment [26, 27, 60, 61]. We have demonstrated that the protective effects of H-MW-HA correlated with a significant reduction in mucosal apoptotic cells and COX-2 expression. Elevated levels of the cytokine IL-6 and the chemokine CXCL1, which are also associated with oral mucositis in humans [11, 37, 39], were found in the murine model after 5-FU treatment and were normalized by H-MW-HA. Interestingly, overexpression of the anti-inflammatory cytokine IL-13, which is known to be significantly elevated in intestinal mucositis [40], was also observed in 5-FU treated mice but was not affected by H-MW-HA treatment. This suggests that H-MW-HA supplementation is particularly efficacious in OM but could be less specific for intestinal inflammation. Finally, superoxide dismutase (SOD), which prevents hydroxyl radicals formation [41], was shown to be significantly elevated in animals treated with both 5-FU and H-MW-HA. We therefore concluded that the protective effect of the H-MW-HA on 5-FU-induced damage may be at least partially attributed to its influence on this antioxidant enzyme activity.

To our knowledge, this is the first study to evaluate the effects of administration of H-MW-HA on the inflammatory, apoptotic and functional aspects of 5-FU-induced OIM. These results provide an experimental rationale to develop a novel HA-derived treatment as a therapeutic agent to protect against apoptotic toxicity associated with anticancer therapy. The use of HA alone or in combination with anticancer agents (5-fluorouracil, doxorubicin, methotrexate, imatinib, gemcitabine, cisplatin, etc.) could be a promising novel pathway for antitumor therapeutics [62–66]. These observations also allow us to speculate that H-MW-HA may not influence the antitumor effect of 5-FU, but this remains to tested in further studies.

## Conclusion

The present work provides unequivocal evidence that H-MW-HA can both prevent and reverse murine tongue and small intestine 5-FU-induced damage. H-MW-HA co-treatment resulted in recovery of jejunum villus length, and tongue epithelial thickness by suppressing both oxidative stress and apoptosis resulting in a reduction of inflammatory mediators. Based on these findings, we conclude that H-MW-HA can efficiently ameliorate 5-FU-induced OIM.

## Materials and methods

### Cell line and culture conditions

To evaluate the protective effects of HA derivatives in an oxidative stress-induced model of oral mucosal injury in vitro, immortalized normal human oral keratinocytes (OKF6) cultures were used as a cellular model of oral mucosa. OKF6 [35] is a benign human oral keratinocyte cell line which expresses hTERT and bypasses a p16^INK4a^ cell cycle control mechanism and derived from the floor of the mouth, provided by Oral Health Cooperative Research Centre (OHCRC), The University of Melbourne, Australia (underwent karyotype analysis prior to experimentation,VCGS Cytogenetics Laboratory, Royal Children’s Hospital, Parkville, VIC). Cells were cultured as discribed elswhere [67]. Briefly, OKF6 cells were cultured as adherent cultures in 100-mm tissue culture-treated plates and grown in keratinocyte serum-free medium (K-SFM) (#17005-042, Thermo Fisher Scientific) supplemented with 25 μg/mL pituitary bovine extract (PBE), 0.2 ng/mL epidermal growth factor (EGF), 1% (v/v) Newborn Calf Serum (NCS) (#N4637, Sigma-Aldrich), 100 IU/ml penicillin, and 100 μg/ml streptomycin (#P4333, Sigma-Aldrich, Castle Hill, NSW, Australia), and containing 0.4 mM calcium chloride CaCl_2_. Cells were grown in a humidified atmosphere at standard conditions (5% CO_2_ at 37 °C).

### HA constituents

The tested compounds included; Mucosamin^®^ spray (Errekappa Euroterapici Spa, Milan, Italy) a commercially available adjuvant gel composed of 1.33% (w/v) HA with a molecular mass of 600–800 kDa, not crosslinked, in combination with a pool of collagen precursor synthetic amino acids, namely L-proline (0.75% w/v), L-leucine (0.15% w/v), L-lysine (0.1% w/v), and glycine (1% w/v); A Natural non-sulphated high molecular weight hyaluronic acid (H-MW-HA) powder (Hyaluronic acid sodium salt derived from Streptococcus equi), non-crosslinked with a molecular weight of ~1.5–1.8 × 10E6 Da, (Cat#53747, Sigma-Aldrich); and three cross-linked (xl-) HA-derivative powders (xl-HA (5/5); EDC/HOBT 5/5 mmol/mL, xl-HA (30/30); EDC/HOBT 30/30 mmol/mL, and xl-HA (100/100); EDC/HOBT, 100/100 mmol/mL). Crosslinked HA derivatives were kindly provided by Dr Annalisa La Gatta (Department Experimental Medicine, Section of Biotechnology, Medical Histology and Molecular Biology, School of Medicine, University of Campania "Luigi Vanvitelli", Naples, Italy).

### In vitro model of oxidative stress-induced toxicity

An in vitro oxidative stress-induced oral mucosa toxicity model was established in OKF6 cells using H_2_O_2_, a well-known inducer of oxidative stress [68, 69]. An initial range of concentrations (100–1200 μM) tested over the given timepoints was determined based on existing literature [28, 70–73]. A series of preliminary experiments were performed to identify a concentration of H_2_O_2_ that inhibited 50% viability (IC_50_) of OKF6 cells at 24 h pre-incubation.

### Treatment of OKF6 cells with HA constituents

OKF6 cells were seeded at density of 1.5 × 10^4^ cells/well in a dark, clear-bottom 96-well microplate (#CLS3603, Sigma-Aldrich) in complete culture medium and incubated overnight at standard conditions to permit for cell attachment. Cell viability was confirmed > 90% by trypan blue exclusion (trypan blue dye, 0.4% solution, 1450021, Bio-Rad). Cells were then pre-treated with 100 µl/well of HA constituents prepared in complete culture medium for 24 hours to allow for HA metabolism and chemical transformation in cells [74, 75]. Pre-treated cells were then co-treated with 100 µl/well of HA constituents with or without 400 µM H_2_O_2_ prepared in fresh complete culture medium and incubated for 24 hours under standard conditions. Concentrations of HA constituents used; Mucosamin^®^, H-MW-HA (1% and 5%, v/v), xl-HA 5/5, xl-HA 30/30, and xl-HA 100/100 (0.01% v/v) and xl-HA (100/100). Concentrations of all HA constituents were chosen for potential safe efficacy, as determined by preliminary experiments (data not shown) and potential therapeutic efficacy of HA-based compound (Mucosamin^®^) [28, 76]. To determine the potential protective effect of HA constituents on H_2_O_2_-induced damage in oral keratinocyte cells, cell viability was assessed using fluorescein diacetate (FDA) fluorescent assay [77]. The FDA fluorescence intensity was quantified at the respective excitation and emission wavelength of 485/20 nm and 528/20 nm using Synergy HTX Multi-Mode Reader (Bio-Tek, USA). Cell viability was expressed as a percentage of fluorescence intensity. Viability of control cells (without treatment) was considered as 100%. The value of cells incubated with the various treatments was compared to control cells. Data are expressed as mean percentage viability relative to the unexposed control (100%). Intracellular ROS production was measured in OKF6 cells by using the cell-permeant 5-(and-6)-chloromethyl-2',7'-dichlorodihydrofluorescein diacetate (CM-H_2_DCFDA, Invitrogen, Thermo Fisher Scientific, C6827), as per manufacturers' instruction. To determine a time course for intracellular ROS generation, kinetic increases in fluorescence of CM-DCF were measured at 0, 15, 30, and 45 min and at 30 min intervals thereafter for a total of 6 h using Synergy HTX Multi-Mode Reader (Bio-Tek, USA). ROS generation of control vehicle (1 unit), and *ROS* formation was measured as the relative change in CM-DCF fluorescence (-fold increase) relative to the control vehicle.

### Animals

C57BL/6 female mice aged 6–12 weeks and weighing between 15 and 20 g were used in this study. Animals were purchased from Bio21 animal facility (Bio21 institute, The University of Melbourne, Parkville, Victoria, Australia). Mice were housed in groups of three or four in clear cages with wood chips under specific pathogen-free conditions and maintained on a 12-hour light/dark cycle in a temperature-controlled and humidity-controlled room (21 ± 1 ˚C and 40%–60%, respectively) with free access to an autoclaved food pellets and water provided ad libitum. This study was approved by the Animal Ethics Committee (AEC) of The University of Melbourne (ID 1814636.3). All work was conducted in compliance with the Australian Code for the Care and Use of Animals for Scientific Purposes determined by the National Health and Medical Research Council 8th edition (2013) [78], and reported according to the ARRIVE guidelines.

### Preparation of 5-fluorouracil (5-FU) and hyaluronic acid (HA)

5-FU powder (Sigma-Aldrich, Castle Hill, NSW, Australia Cat#F6627) was dissolved in saline to a final concentration of 10 mg/ml. A Natural non-sulfated high molecular weight hyaluronic acid (H-MW-HA) powder (non-crosslinked, mol wt ~1.5–1.8 × 10E6 Da, Hyaluronic acid sodium salt, from Streptococcus equi, Cat#53747, Sigma-Aldrich, Castle Hill, NSW, Australia) was dissolved in drinking water at a concentration of 0.01% (w/v).

### Induction of chemotherapy-induced oral and intestinal mucositis (CIOIM)

Mice were induced to develop CIOIM as previously described [37]. Briefly, a total of sixty mice were used in the study. The mice were randomly divided into four experimental groups (Supplementary Fig. S4); A, B, C, and D, with respectively *n* = 12, 12, 18, and 18 mice per group: A (control, *n* = 12). B (H-MW-HA only, *n* = 12), mice received H-MW-HA (0.01% w/v) orally in drinking water from day 0 until experimental endpoint. C (5-FU, *n* = 18), mice received 5-FU injection i.v. (50 mg/kg/day) every 48 h, from day 1 to day 13. D (5-FU + H-MW-HA, *n* = 18), mice received a 5-FU i.v. injection (50 mg/kg/day), every 48 hours, from day 1 to day 13, plus HA-MW-HA (0.01% w/v) orally in drinking water from day 0 until experimental endpoint. Control and H-MW-HA groups received physiological saline i.v (vehicle). In all cases i.v. drug administration was performed via tail vein injections. Mice were monitored daily for signs of morbidity, with body weights recorded every 24–48 h.

### Assessment of oral mucositis

Over the 14, 16, or 19 day observation period, mice were anesthetized with isoflurane every 48 h, starting from day 2 until day 10, then every 24 h until the end of the experiment for oral cavity inspection, which included the anteroventral and dorsal tongue, palate, floor of the mouth, lips, and right and left buccal mucosa. Mice were additionally examined for mucositis/ulcer formation and general appearance by macroscopic means by using specialized tailored oral cavity diagnostic tools (Supplementary Fig. S5A and B). Incidence, development, and extent of oral mucositis were assessed clinically in vivo using a visual oral ulcerative mucositis score, adapted from Nakajima et al. [79], based on a modification of the method of Sonis et al. [80] (Supplementary Table S6). Image acquisition was recorded digitally (Canon EOS 60D; Canon Inc., Tokyo, Japan) using a 100 mm lens (Canon 100 mm f/2.8 Macro USM lens) connected with a ring flash (Sigma EM-140 DG Ring Flash).

### Diarrhea assessment

The severity of 5-FU-induced gastrointestinal mucositis was assessed by scoring of stool passages (diarrhea assessment). Stool passages/diarrhea severity of all mice were recorded every 48 h, starting from day 2 post initial injection, until day 10, and then every 24 h until the end of the experiment. Diarrhea severity was assessed using Bowen’s score system [81] and classified into four grades according to the stool consistency: 0, normal stool; 1, slightly wet and soft stool indicating mild diarrhea; 2, wet and unformed stool indicating moderate diarrhea; 3, watery stool indicating severe diarrhea.

### Macroscopic examination of the tongue

Animals were sacrificed on days 14, 16, and 19. Blood samples were obtained and serum frozen until analysis. At necropsy, the tongue was excised at the level of the trachea. To reveal surface erosive or ulcerative lesions, the tongue was stained with 1% toluidine blue in 10% acetic acid for 1 minute, followed by repeated washes with acetic acid until no further recovery of dye was evident [82]. Tongues images were acquired with a digital camera (Canon EOS 60D; Canon Inc., Tokyo, Japan) using 100 mm lens (Canon 100 mm f/2.8 Macro USM lens) connected with a ring flash (Sigma EM-140 DG Ring Flash) and scored according to the following criteria; Negative—lack of dye uptake or light, diffusely stippled uptake of dye; positive—deep, royal blue staining of the epithelium and lack of epithelium (identified as ulcer). The percentage of toluidine blue positive surface area (excluding excision trauma) was calculated using Fiji (ImageJ) software [83, 84].

### Histopathologic examination

At necropsy, jejunum and tongue specimens were collected, and immersed in 10% neutralized formalin overnight. The tissues were embedded in paraffin, cut into 4-µm-thick sections, and stained with hematoxylin and eosin (H&E). Slides were scanned as digital images using Olympus VS120 automated slide scanner equipped with a BX61VS microscope with a 20× objective (Olympus VS120-S6-W, Olympus VS-ASW software). Scanned digital images of photomicrographs were examined using QuPath open source digital software v. 0.2.0 [85]. For the morphometric analysis of the jejunum intestinal wall, tissue samples were oriented with longitudinally cut villi to assess tunica mucosa thickness, intestinal villi length and crypt depth (Supplementary Fig. S5C). Analysis was conducted as described by Navarrete et al. [86]. Briefly, three mice per group were analyzed, with the analysis consisting of three intestinal rings and one longitudinal intestinal section per mouse. Each analysis incorporated 3 fields per section and 1 measurement per field, with 12 intact villi and crypts measured and averaged. For the morphometric analysis of the tongue, epithelial tongue thickness was measured as described by Carrard et al. [87]. Briefly, epithelial thickness was measured from the basal membrane to the granular layer. Three mice per group, and three transverse sections per tongue, consisting of 3 fields per section and 3 measurements per field, were measured and averaged (Supplementary Fig. S5D). Scores were allocated in a blinded manner throughout.

### Immunohistochemistry

Formalin fixed, paraffin embedded (FFPE) tongue and jejunum samples were sectioned onto Superfrost slides (Thermo Fisher Scientific, MA, USA) then dewaxed and rehydrated according to standard protocols. Slides were then prepared for IHC with anti-COX-2 (SP21, 1/50, Thermofisher# MA5-14568.) or anti-cleaved -caspase-3 (CC3) (#9661 (Ap175), 1/300, Cell Signalling Technology; MA, USA) polyclonal antibodies using an Autostainer (Dako Omnis, Agilent Technologies, Vic., Australia). Sections were first subjected to heat-induced antigen retrieval with EnVision FLEX TRS, low pH (cleaved caspase-3 (CC3)) or high pH (COX-2) at 97 ^o^C and washed. Sections to be stained for CC3 were then incubated with blocking reagents (X0590 Avidin stock, X0590 biotin stock (each 10 min, (Dako)), followed by FLEX peroxidase blocking agent (5 min). Incubation with primary antibodies then followed (45–60 min, COX-2 and CC3 respectively) with post-washing. CC3 staining was detected with anti-Rabbit biotin (1/300, Dako) and ABC kit (Dako) incubations and COX-2 by rabbit-labeled polymer-HRP (Dako k4003) followed by EnV FLEX substrate solution (10 min), washing and counterstaining according to standard protocols.

TUNEL staining was performed as before [88], with terminal deoxynucleotide transferase (Promega# M828C) followed by Biotin-16-dUTP (Roche #11093070910) according to the manufacturers’ instructions. All slides were scanned as digital images using Olympus VS120 automated slide scanner equipped with a BX61VS microscope with a 20× objective (Olympus VS120-S6-W, Olympus VS-ASW software). Scanned digital images were examined using QuPath open source digital software v. 0.2.0 [85]. Histopathology was assessed in blinded fashion for numbers of apoptotic cells and COX-2 positive cells.

### Multiplex analysis of serum cytokine and chemokine levels

Serum cytokine and chemokine levels were measured using the Bio-Plex Pro™ mouse cytokine magnetic bead immunoassay (23-plex; Bio-Rad# M60009RDPD) on a Bio-Rad Bio-Plex instrument, following the manufacturer’s instructions as before [88]. The cytokines measured include IL-1Α, IL-Β, IL-2, IL-3, IL-4, IL-5, IL-6, IL-9, IL-10, IL-12(P40), IL-12(p70), IL-13, IL-17A, Eotaxin, G-CSF, GM-CSF, IFNG, KC (CXCL1), MCP-1, MIP-1Α, MIP-1Β, RANTES, and TNF α.

### Superoxide dismutase (SOD) enzyme activity assay

Total superoxide dismutase activity was measured in blood serum using an indirect enzyme assay (SOD Assay Kit, 19160, Sigma Aldrich, Castle Hill, NSW, Australia), according to the manufacturer’s instructions. Briefly, SOD activity was assayed spectrophotometrically (Perkin Wallac Victor 2 V Multilabel Microplate Reader 1420, Shelton, USA) by absorbance measurement at 450 nm. Samples were run in triplicate and values expressed as U/ml of blood.

### Statistics

Statistical analysis was performed using IBM SPSS Statistics for Windows, version 42 (IBM Corp., Armonk, N.Y., USA) and GraphPad Prism version 8.0.1 for windows (GraphPad Software Inc, San Diego, CA). Data is presented as means ± SD, unless otherwise mentioned, and evaluated by One-way analysis of variance ANOVA followed by Tukey’s post hoc test to detect inter-group differences. Pearson chi-square tests for independence (categorical variables) were used to examine differences in ulcer status and diarrheal status between different treatment groups. Fit spline/LOWESS test was used to calculate IC50. Logrank test was used for comparative analysis of survival rates. Differences of *p* < 0.05 were considered to be statistically significant.

## Supplementary information

Supplementary Material

Reproducibility checklist

## Data Availability

Complete dataset for this study will be made available upon reasonable request to the corresponding authors.

## References

[1] Scully C, Sonis S, Diz PD (2006). Oral mucositis. Oral Dis.

[2] Pulito C, Cristaudo A, Porta CL, Zapperi S, Blandino G, Morrone A (2020). Oral mucositis: the hidden side of cancer therapy. J Exp Clin Cancer Res.

[3] Dahlgren D, Sjöblom M, Hellström PM, Lennernäs H (2021). Chemotherapeutics-induced intestinal mucositis: pathophysiology and potential treatment strategies. Front Pharmacol.

[4] Pico JL, Avila‐Garavito A, Naccache P (1998). Mucositis: its occurrence, consequences, and treatment in the oncology setting. Oncologist.

[5] Keefe D, Brealey J, Goland G, Cummins A (2000). Chemotherapy for cancer causes apoptosis that precedes hypoplasia in crypts of the small intestine in humans. Gut..

[6] Keefe DM (2004). Gastrointestinal mucositis: a new biological model. Supportive Care Cancer.

[7] Lalla RV, Peterson DE (2006). Treatment of mucositis, including new medications. Cancer J.

[8] da Cruz Campos MI, Neiva Campos C, Monteiro Aarestrup F, Aarestrup V, Julião B (2014). Oral mucositis in cancer treatment: natural history, prevention and treatment. Mol Clin Oncol.

[9] Sonis ST, Elting LS, Keefe D, Peterson DE, Schubert M, Hauer-Jensen M (2004). Perspectives on cancer therapy-induced mucosal injury: pathogenesis, measurement. Epidemiol, Conséq Patients Cancer.

[10] Al-Dasooqi N, Sonis ST, Bowen JM, Bateman E, Blijlevens N, Gibson RJ (2013). Emerging evidence on the pathobiology of mucositis. Support Care Cancer.

[11] Villa A, Sonis ST (2015). Mucositis: pathobiology and management. Curr Opin Oncol.

[12] Lalla RV, Bowen J, Barasch A, Elting L, Epstein J, Keefe DM (2014). MASCC/ISOO clinical practice guidelines for the management of mucositis secondary to cancer therapy. Cancer..

[13] Grem JL (2000). 5-Fluorouracil: forty-plus and still ticking. A review of its preclinical and clinical development. investig. N. Drugs.

[14] Curra M, Junior S, Valente LA, Martins MD, Santos PSDS (2018). Chemotherapy protocols and incidence of oral mucositis. Integr Rev Einstein.

[15] Brown CG, Wingard J (2004). Clinical consequences of oral mucositis. Semin Oncol Nurs.

[16] Yuan A, Sonis S (2014). Emerging therapies for the prevention and treatment of oral mucositis. Expert Opin Emerg Drugs.

[17] Carlotto A, Hogsett VL, Maiorini EM, Razulis JG, Sonis ST (2013). The economic burden of toxicities associated with cancer treatment: review of the literature and analysis of nausea and vomiting, diarrhoea, oral mucositis and fatigue. Pharmacoeconomics..

[18] Sonis ST (2011). Oral mucositis. Anticancer Drugs.

[19] Colella G, Cannavale R, Vicidomini A, Rinaldi G, Compilato D, Campisi G (2010). Efficacy of a Spray Compound Containing a Pool of Collagen Precursor Synthetic Amino Acids (L-Proline, L-Leucine, L-Lysine and Glycine) Combined with Sodium Hyaluronate to Manage Chemo/Radiotherapy-Induced Oral Mucositis: Preliminary Data of an Open Clinical Trial. Int J Immunopathol Pharmacol.

[20] Cohen EEW, Ahmed O, Kocherginsky M, Shustakova G, Kistner-Griffin E, Salama JK (2013). Study of functional infrared imaging for early detection of mucositis in locally advanced head and neck cancer treated with chemoradiotherapy. Oral Oncol.

[21] Raber-Durlacher JE, von Bültzingslöwen I, Logan RM, Bowen J, Al-Azri AR, Everaus H (2013). Systematic review of cytokines and growth factors for the management of oral mucositis in cancer patients. Supportive Care Cancer.

[22] Nicolatou-Galitis O, Sarri T, Bowen J, Di Palma M, Kouloulias VE, Niscola P (2013). Systematic review of anti-inflammatory agents for the management of oral mucositis in cancer patients. Supportive Care Cancer.

[23] Stokman MA, Spijkervet FKL, Boezen HM, Schouten JP, Roodenburg JLN, de Vries EGE (2006). Preventive intervention possibilities in radiotherapy- and chemotherapy-induced oral mucositis: results of meta-analyses. J Dent Res.

[24] Duncan M, Grant G (2003). Oral and intestinal mucositis—causes and possible treatments. Alimentary Pharmacol Therapeutics.

[25] Campos MIDC, Campos CN, Aarestrup FM, Aarestrup BJV (2014). Oral mucositis in cancer treatment: natural history, prevention and treatment (review). Mol Clin Oncol.

[26] Criswell T, Leskov K, Miyamoto S, Luo G, Boothman DA (2003). Transcription factors activated in mammalian cells after clinically relevant doses of ionizing radiation. Oncogene..

[27] Yoshino F, Yoshida A, Nakajima A, Wada-Takahashi S, Takahashi S-S, Lee MC-I (2013). Alteration of the redox state with reactive oxygen species for 5-fluorouracil-induced oral mucositis in hamsters. PLoS ONE.

[28] Cirillo N, Vicidomini A, McCullough M, Gambardella A, Hassona Y, Prime SS (2015). A hyaluronic acid-based compound inhibits fibroblast senescence induced by oxidative stress in vitro and prevents oral mucositis in vivo. J Cell Physiol.

[29] Rah MJ (2011). A review of hyaluronan and its ophthalmic applications. Optometry.

[30] David‐Raoudi M, Tranchepain F, Deschrevel B, Vincent JC, Bogdanowicz P, Boumediene K (2008). Differential effects of hyaluronan and its fragments on fibroblasts: relation to wound healing. Wound Repair Regeneration.

[31] Favia G, Mariggio MA, Maiorano F, Cassano A, Capodiferro S, Ribatti D (2008). Accelerated wound healing of oral soft tissues and angiogenic effect induced by a pool of aminoacids combined to sodium hyaluronate (AMINOGAM). J Biol Regulators Homeost Agents.

[32] Buchsel PC (2008). Polyvinylpyrrolidone-sodium hyaluronate gel (Gelclair): a bioadherent oral gel for the treatment of oral mucositis and other painful oral lesions. Expert Opin Drug Metab Toxicol.

[33] Ruggiero T, Pol R, Camisassa D, Arata V, Martino I, Giaccone L (2016). Use of sodium hyaluronate and synthetic amino acid precursors of collagen for the symptomatic treatment of mucositis in patients undergoing haematopoietic stem cell transplants. J Biol Regul Homeost Agents.

[34] Colella G, Vicidomini A, Soro V, Lanza A, Cirillo N (2012). Molecular insights into the effects of sodium hyaluronate preparations in keratinocytes. Clin Exp Dermatol.

[35] Dickson MA, Hahn WC, Ino Y, Ronfard V, Wu JY, Weinberg RA (2000). Human keratinocytes that express hTERT and also bypass a p16INK4a-enforced mechanism that limits life span become immortal yet retain normal growth and differentiation characteristics. Mol Cell Biol.

[36] Mohammed AI, Celentano A, Paolini R, Low JT, McCullough MJ, O’Reilly LA (2023). Characterization of a novel dual murine model of chemotherapy-induced oral and intestinal mucositis. Sci Rep..

[37] Bertolini M, Sobue T, Thompson A, Dongari-Bagtzoglou A (2017). Chemotherapy induces oral mucositis in mice without additional noxious stimuli. Transl Oncol.

[38] Sobue T, Bertolini M, Thompson A, Peterson DE, Diaz PI, Dongari‐Bagtzoglou A (2018). Chemotherapy‐induced oral mucositis and associated infections in a novel organotypic model. Mol Oral Microbiol.

[39] Cinausero M, Aprile G, Ermacora P, Basile D, Vitale MG, Fanotto V (2017). New frontiers in the pathobiology and treatment of cancer regimen-related mucosal injury. Front Pharmacol.

[40] Gan Y, Ai G, Wu J, Luo H, Chen L, Huang Q (2020). Patchouli oil ameliorates 5-fluorouracil-induced intestinal mucositis in rats via protecting intestinal barrier and regulating water transport. J Ethnopharmacol.

[41] Cui J-J, Yuan J-F, Zhang Z-Q (2010). Anti-oxidation activity of the crude polysaccharides isolated from Polygonum cillinerve (Nakai) Ohwi in immunosuppressed mice. J Ethnopharmacol.

[42] Cheng KKF, Lee V, Li CH, Yuen HL, Epstein JB (2012). Oral mucositis in pediatric and adolescent patients undergoing chemotherapy: the impact of symptoms on quality of life. Supportive Care Cancer.

[43] Elting LS, Cooksley C, Chambers M, Cantor SB, Manzullo E, Rubenstein EB (2003). The burdens of cancer therapy: clinical and economic outcomes of chemotherapy‐induced mucositis. Cancer: interdisciplinary. Int J Am Cancer Soc.

[44] Sonis ST, Oster G, Fuchs H, Bellm L, Bradford WZ, Edelsberg J (2001). Oral mucositis and the clinical and economic outcomes of hematopoietic stem-cell transplantation. J Clin Oncol.

[45] Sougiannis AT, VanderVeen BN, Davis JM, Fan D, Murphy EA (2021). Understanding chemotherapy-induced intestinal mucositis and strategies to improve gut resilience. Am J Physiol-Gastrointest Liver Physiol.

[46] Elad S, Yarom N, Zadik Y, Kuten-Shorrer M, Sonis ST (2022). The broadening scope of oral mucositis and oral ulcerative mucosal toxicities of anticancer therapies. CA Cancer J Clin.

[47] Stein A, Voigt W, Jordan K (2010). Chemotherapy-induced diarrhea: pathophysiology, frequency and guideline-based management. Therapeutic Adv Med Oncol.

[48] Huang J, Hwang AYM, Jia Y, Kim B, Iskandar M, Mohammed AI (2022). Experimental chemotherapy-induced mucositis: a scoping review guiding the design of suitable preclinical models. Int J Mol Sci.

[49] Koselke E, Kraft S (2012). Chemotherapy-induced diarrhea: options for treatment and prevention. J Hematol Oncol Pharmacy.

[50] Al-Asmari AK, Khan AQ, Al-Qasim AM, Al-Yousef Y (2015). Ascorbic acid attenuates antineoplastic drug 5-fluorouracil induced gastrointestinal toxicity in rats by modulating the expression of inflammatory mediators. Toxicol Rep..

[51] Chen C, Tian L, Zhang M, Sun Q, Zhang X, Li X (2013). Protective effect of amifostine on high-dose methotrexate-induced small intestinal mucositis in mice. Digestive Dis Sci.

[52] Justino PF, Melo LF, Nogueira AF, Costa JV, Silva LM, Santos CM (2014). Treatment with Saccharomyces boulardii reduces the inflammation and dysfunction of the gastrointestinal tract in 5-fluorouracil-induced intestinal mucositis in mice. Br J Nutr.

[53] Montagner IM, Merlo A, Carpanese D, Zuccolotto G, Renier D, Campisi M (2015). Drug conjugation to hyaluronan widens therapeutic indications for ovarian cancer. Oncoscience..

[54] Banzato A, Bobisse S, Rondina M, Renier D, Bettella F, Esposito G (2008). A paclitaxel-hyaluronan bioconjugate targeting ovarian cancer affords a potent in vivo therapeutic activity. Clin Cancer Res.

[55] Kim H, Park HT, Tae YM, Kong WH, Sung DK, Hwang BW (2013). Bioimaging and pulmonary applications of self-assembled Flt1 peptide–hyaluronic acid conjugate nanoparticles. Biomaterials..

[56] Lee H, Kim JB, Park SY, Kim SS, Kim H (2013). Combination effect of paclitaxel and hyaluronic acid on cancer stem-like side population cells. J Biomed Nanotechnol.

[57] Mero A, Campisi M (2014). Hyaluronic acid bioconjugates for the delivery of bioactive molecules. Polymers..

[58] Chen Y, Peng F, Song X, Wu J, Yao W, Gao X (2018). Conjugation of paclitaxel to C-6 hexanediamine-modified hyaluronic acid for targeted drug delivery to enhance antitumor efficacy. Carbohydr Polym.

[59] Lamson DW, Brignall M (1999). Antioxidants in cancer therapy; their actions and interactions with oncologic therapies. Alternative Med Rev.

[60] Soares PMG, Mota JMSC, Gomes AS, Oliveira RB, Assreuy AMS, Brito GAC (2008). Gastrointestinal dysmotility in 5-fluorouracil-induced intestinal mucositis outlasts inflammatory process resolution. Cancer Chemother Pharmacol.

[61] Kim HJ, Kim JH, Moon W, Park J, Park SJ, Song GA (2015). Rebamipide attenuates 5-fluorouracil-induced small intestinal mucositis in a mouse model. Biol Pharm Bull.

[62] Toole BP, Ghatak S, Misra S (2008). Hyaluronan oligosaccharides as a potential anticancer therapeutic. Curr Pharm Biotechnol.

[63] Seton-Rogers S (2012). Multitasking hyaluronic acid. Nat Rev Cancer.

[64] Lokeshwar VB, Mirza S, Jordan A. Targeting hyaluronic acid family for cancer chemoprevention and therapy. Adv Cancer Res. 2014;123:35–65. 10.1016/B978-0-12-800092-2.00002-2 PMC4791948 25081525

[65] Negi LM, Jaggi M, Joshi V, Ronodip K, Talegaonkar S (2015). Hyaluronan coated liposomes as the intravenous platform for delivery of imatinib mesylate in MDR colon cancer. Int J Biol Macromol.

[66] Han N-K, Shin DH, Kim JS, Weon KY, Jang C-Y, Kim J-S (2016). Hyaluronan-conjugated liposomes encapsulating gemcitabine for breast cancer stem cells. Int J Nanomed.

[67] Yiannis C, Huang K, Tran AN, Zeng C, Dao E, Baselyous O (2020). Protective effect of kava constituents in an in vitro model of oral mucositis. J Cancer Res Clin Oncol.

[68] Gille J, Joenje H. Cell culture models for oxidative stress: superoxide and hydrogen peroxide versus normobaric hyperoxia. Mutation Research/DNAging. 1992;275:405–14. 10.1016/0921-8734(92)90043-o 1383781

[69] Cross CE, Tolba MF, Rondelli CM, Xu M, Abdel-Rahman SZ (2015). Oxidative Stress Alters miRNA and Gene Expression Profiles in Villous First Trimester Trophoblasts. Biomed Res Int.

[70] Lin K-Y, Chung C-H, Ciou J-S, Su P-F, Wang P-W, Shieh D-B (2019). Molecular damage and responses of oral keratinocyte to hydrogen peroxide. BMC Oral Health.

[71] Hseu YC, Yang TY, Li ML, Rajendran P, Mathew DC, Tsai CH (2019). Chalcone flavokawain A attenuates TGF‐β1‐induced fibrotic pathology via inhibition of ROS/Smad3 signaling pathways and induction of Nrf2/ARE‐mediated antioxidant genes in vascular smooth muscle cells. J Cell Mol Med.

[72] Man W, Ming D, Fang D, Chao L, Jing C (2014). Dimethyl sulfoxide attenuates hydrogen peroxide‐induced injury in cardiomyocytes via heme oxygenase‐1. J Cell Biochem.

[73] Royack G, Nguyen M, Tong D, Poot M, Oda D (2000). Response of human oral epithelial cells to oxidative damage and the effect of vitamin E. Oral Oncol.

[74] Lee JY, Spicer AP (2000). Hyaluronan: a multifunctional, megaDalton, stealth molecule. Curr Opin Cell Biol.

[75] Lepperdinger G, Fehrer C, Reitinger S. Biodegradation of hyaluronan. Elsevier Press: Amsterdam; 2004.

[76] La Gatta A, D'Agostino A, Schiraldi C, Colella G, Cirillo N (2019). A biophysically-defined hyaluronic acid-based compound accelerates migration and stimulates the production of keratinocyte-derived neuromodulators. Cell Adhes Migr.

[77] Lindhagen E, Nygren P, Larsson R (2008). The fluorometric microculture cytotoxicity assay. Nat Protoc.

[78] Hubrecht R (2013). Revised Australian Code for the care and use of animals for scientific purposes. Anim Welf.

[79] Nakajima N, Watanabe S, Kiyoi T, Tanaka A, Suemaru K, Araki H (2015). Evaluation of edaravone against radiation-induced oral mucositis in mice. J Pharm Sci.

[80] Sonis ST, Peterson RL, Edwards LJ, Lucey CA, Wang L, Mason L (2000). Defining mechanisms of action of interleukin-11 on the progression of radiation-induced oral mucositis in hamsters. Oral Oncol.

[81] Gibson RJ, Bowen JM, Inglis MR, Cummins AG, Keefe DM (2003). Irinotecan causes severe small intestinal damage, as well as colonic damage, in the rat with implanted breast cancer. J Gastroenterol Hepatol.

[82] Muanza TM, Cotrim AP, McAuliffe M, Sowers AL, Baum BJ, Cook JA (2005). Evaluation of radiation-induced oral mucositis by optical coherence tomography. Clin Cancer Res.

[83] Schneider CA, Rasband WS, Eliceiri KW (2012). NIH Image to ImageJ: 25 years of image analysis. Nat Methods.

[84] Schindelin J, Arganda-Carreras I, Frise E, Kaynig V, Longair M, Pietzsch T (2012). Fiji: an open-source platform for biological-image analysis. Nat Methods.

[85] Bankhead P, Loughrey MB, Fernández JA, Dombrowski Y, McArt DG, Dunne PD (2017). QuPath: Open source software for digital pathology image analysis. Sci Rep..

[86] Navarrete J, Vásquez B, del Sol M (2015). Morphoquantitative analysis of the Ileum of C57BL/6 mice (Mus musculus) fed with a high-fat diet. Int J Clin Exp Pathol.

[87] Carrard VC, Pires AS, Badauy CM, Rados PV, Lauxen IS, Sant'Ana Filho M (2008). Effects of aging on mouse tongue epithelium focusing on cell proliferation rate and morphological aspects. Bull Tokyo Dent Coll.

[88] O'Reilly LA, Putoczki TL, Mielke LA, Low JT, Lin A, Preaudet A (2018). Loss of NF-κB1 causes gastric cancer with aberrant inflammation and expression of immune checkpoint regulators in a STAT-1-dependent manner. Immunity..

