# Supplementary material for: High molecular weight hyaluronic acid drastically reduces chemotherapy-induced mucositis and apoptotic cell death
Source: Cell Death Dis. 2023 Jul 21;14(7):453. doi: 10.1038/s41419-023-05934-6 (PMC10362044; doi:10.1038/s41419-023-05934-6)
Supplement: Supplementary file 1 — Supplementary Material [file 41419_2023_5934_MOESM1_ESM.docx]

**Supplementary Figures**


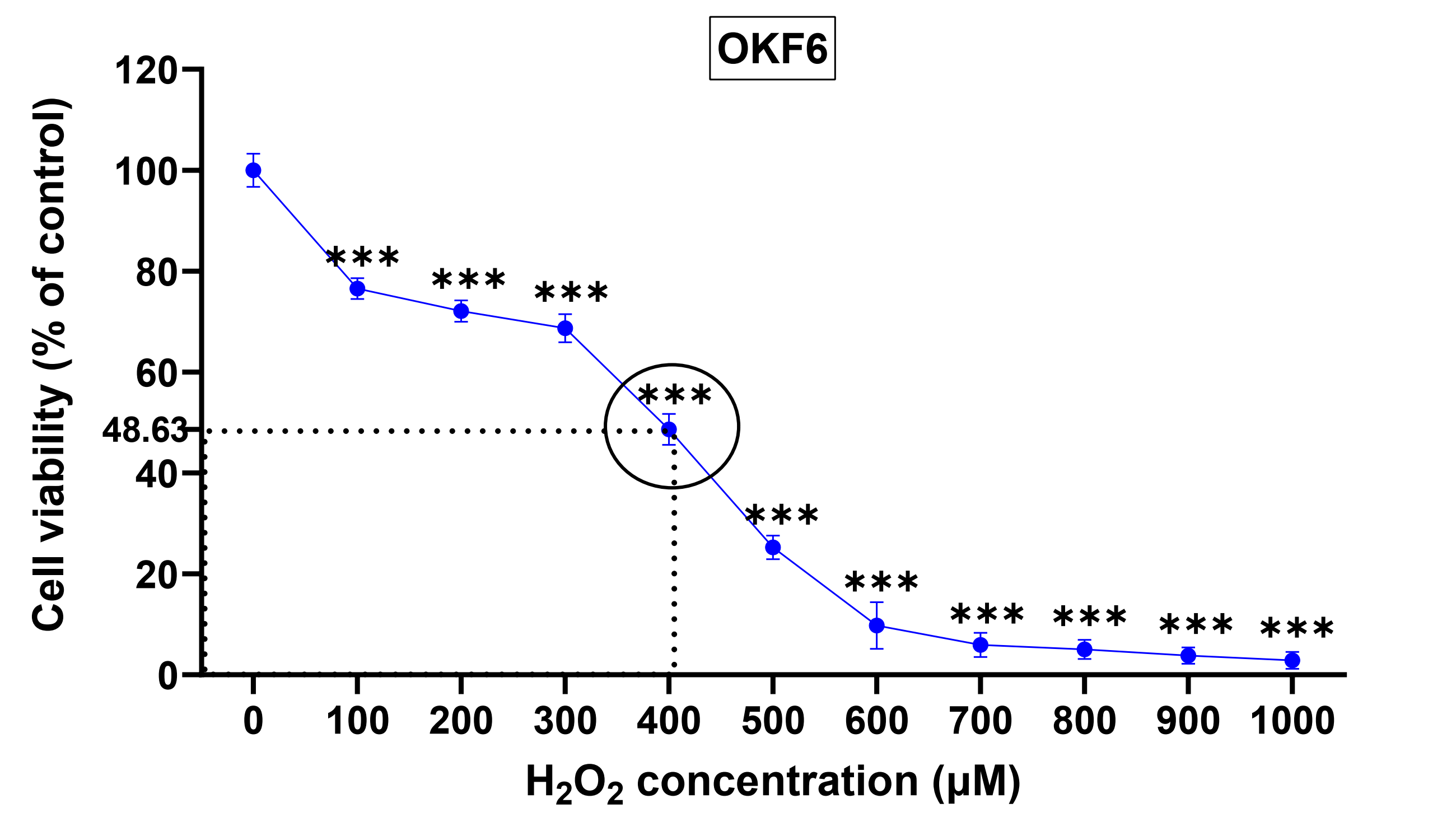


**Figure S1. H_2_O_2_-induced loss of cell proliferation in normal oral epithelial cells.** Immortalized normal human oral keratinocyte (OKF6) cells were seeded at densities of 1.5 x10^4^ in 96-well plates and incubated for 24 hours with increasing concentrations of H_2_O_2_. Cell viability after 24 hours of incubation with H_2_O_2_ was measured by ﬂuorescein diacetate (FDA) ﬂuorescence in a Synergy HTX Multi-Mode Reader (Bio-Tek, USA). The data are expressed as the relative response of treated cells as compared to untreated controls (100%) and represent means ± SD from at least three technical replicates of 3 biological replicate experiments. Statistical significance is given as follows: * p < 0.05, ** p < 0.005, and *** p < 0.005 as compared to untreated controls.


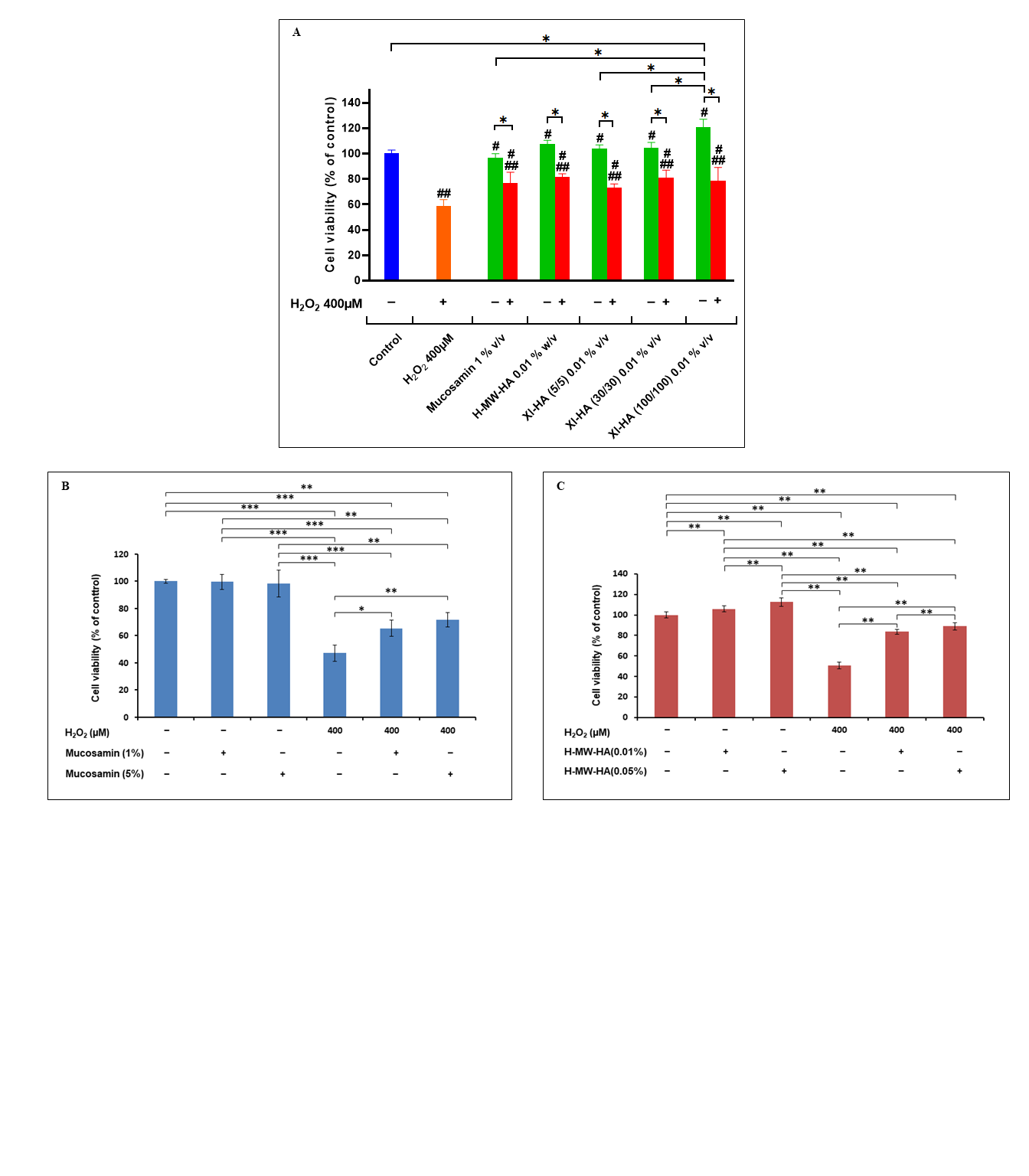


**Figure S2.** **The protective effect of hyaluronic acid (HA) constituents on hydrogen peroxide (H_2_O_2_)-induced cytotoxicity in oral epithelial cells.** OKF6 cells were pre-treated with HA constituents (HA) for 24 hours (Mucosamin^®^ 1% v/v, H-MW-HA 0.01% w/v, Xl-HA 5/5 0.01% v/v, Xl-HA 30/30 0.01% v/v, and Xl-HA 100/100 0.01% v/v) (A), Mucosamin^®^ (1% and 5% v/v) (B), or H-MW-HA (0.01% and 0.05% w/v) (C), and then were co-incubated with the same HA constituents with or without H_2_O_2_ (400 µM) for further 24 hours. At the end of experiment, cell viability was quantified by fluorescein diacetate (FDA) fluorescent assay and expressed as the relative response (%) of treated cells as compared to untreated controls (as control cell viability = 100%). Data are expressed as means ± SD of readings from three wells per data point (technical triplicate). (A) * p < 0.05; ## p < 0.05 as compared to control group; and # p < 0.05 as compared to H_2_O_2_ group. (B) * p < 0.05; ** p < 0.01, *** p < 0.001. (C) * p < 0.005; ** p < 0.001.


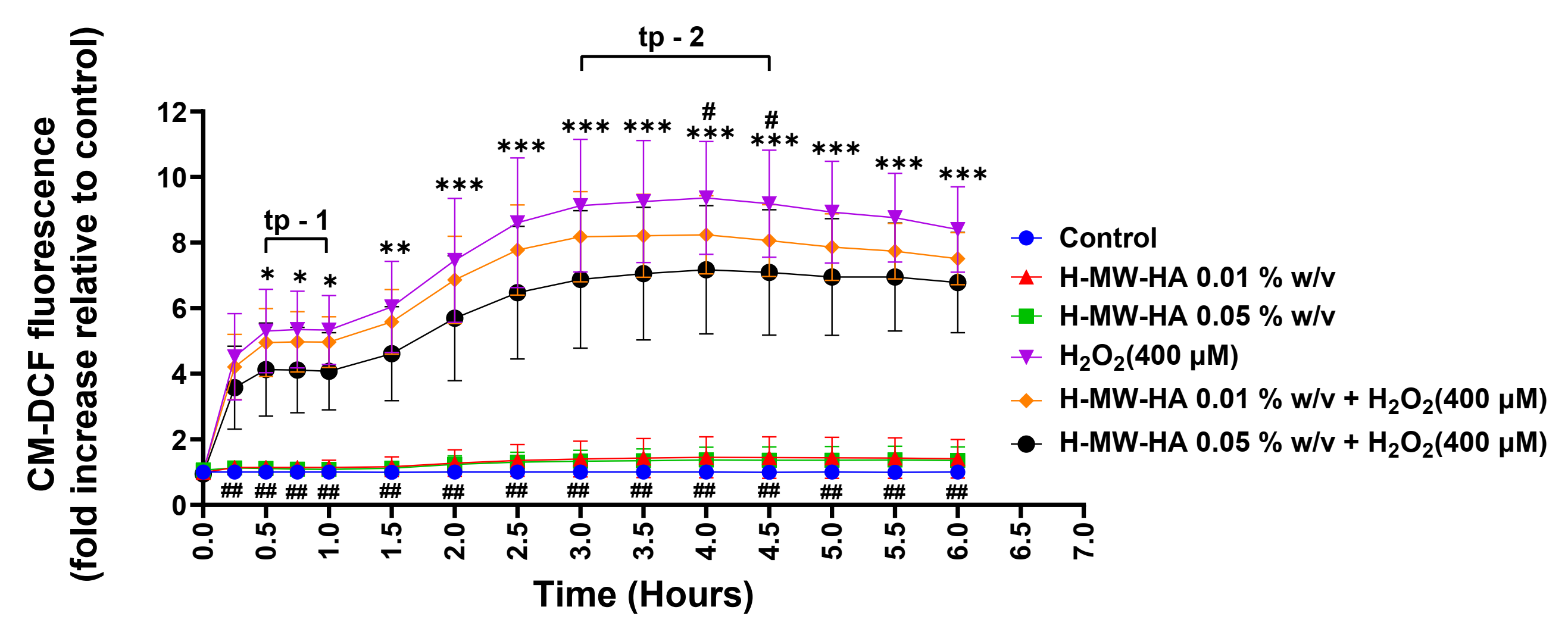


**Figure S3. Protective effect of H-MW-HA (0.01%, and 0.05% w/v) against H_2_O_2_-induced ROS production.** OKF-6 cells were cultured with H-MW-HA (0.01% w/v, and 0.05% w/v) in complete keratinocyte serum free media (K-SFM, #17005-042, Thermo Fisher Scientific) for 24 hours in 96-well culture plates (Cat#: CLS3603; Sigma-Aldrich) under standard conditions (5% CO2 at 37 ° C). Culture medium was then withdrawn and cells were incubated with the fluorescent probe, 5-(and-6)-chloromethyl - 2', 7'- dichlorodihydrofluorescein diacetate (CM-H_2_DCFDA), for 30 minutes. Fluorescent probe was then replaced with complete culture medium supplemented with H-MW-HA (0.01% w/v, and 0.05% w/v) either in the absence or the presence of H_2_O_2_(400 μM). ROS production was measured as the change in CM-DCF fluorescence (-fold increase) relative to control as CRT=1 at timepoints 0, 15 min, 30 min, 45 min, and every 30 min for up to 6 hrs. CM-DCF fluorescence intensity was measured using Synergy HTX Multi-Mode Reader (Bio-Tek, USA) with maximum excitation and emission spectra of 495 nm and 529 nm, respectively. Data are from 3 independent experiments (three biological replicates), n= 4 per group (four technical replicates). Values represent mean ± SD. Where, # p < 0.05; compared to H-MW-HA (0.01% w/v) + H_2_O_2_ (400 μM) treated group, * p < 0.05, ** p < 0.01, *** p < 0.001; compared to H-MW-HA (0.05% w/v) + H_2_O_2_ (400 μM) treated group, and ## p < 0.05 compared to H_2_O_2_ (400 μM) treated group, H-MW-HA (0.01% w/v) + H_2_O_2_ (400 μM) treated group, or H-MW-HA (0.05% w/v) + H_2_O_2_ (400 μM) treated group. Abbreviations: tp-1, time plateau-1; tp-2, time plateau-2.


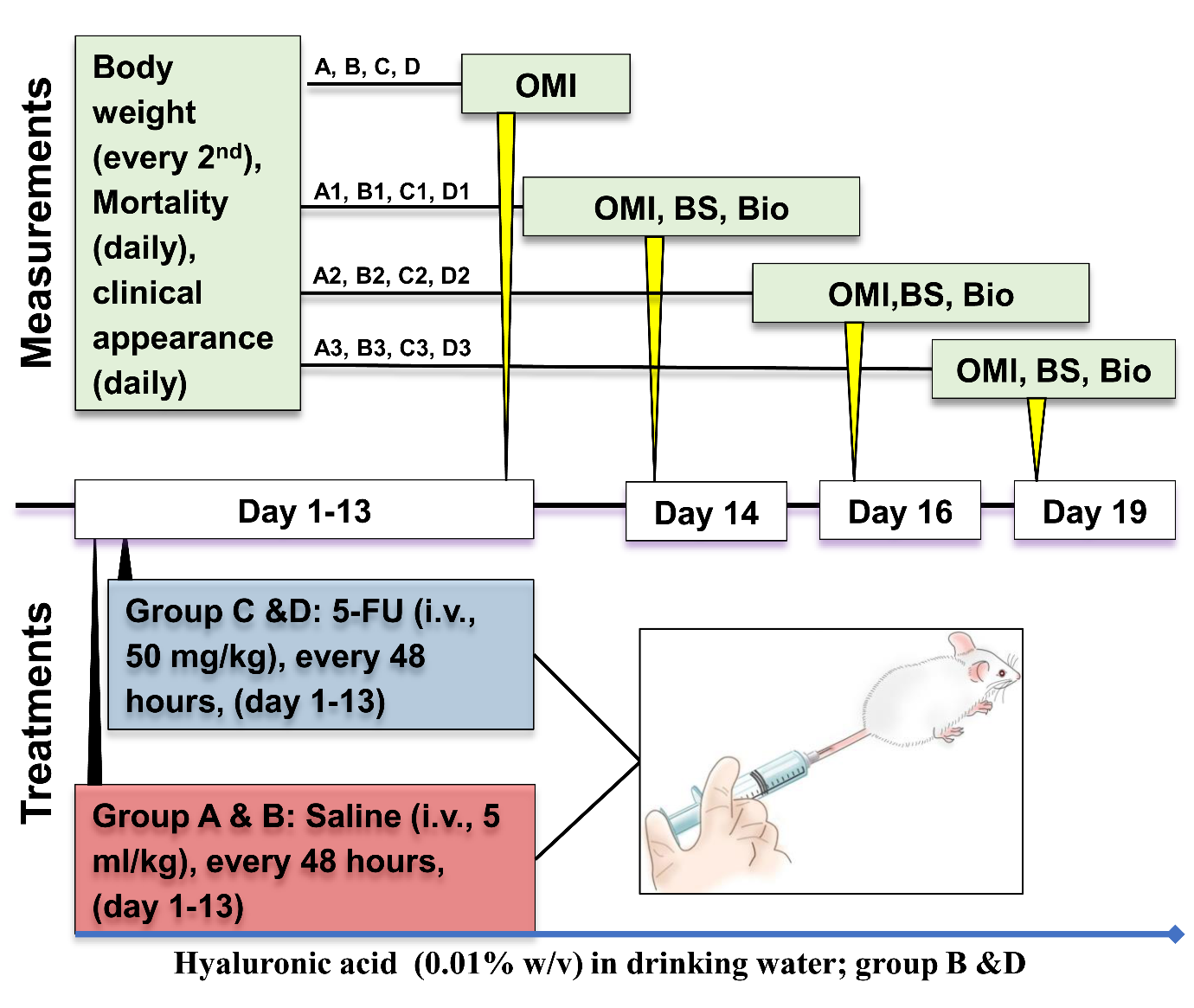


**Figure S4. Establishing 5FU-induced oral and intestinal mice model.** The schematics of experimental procedure. 6-12 weeks-old C57BL/6 female mice were divided into 4 groups and treated as follows: Group A; Saline as normal control; Group B; H-MW-HA treatment only; Group C; 5-FU as positive control; Group D; 5-FU + H-MW-HA. The H-MW-HA treatment was given in drinking water starting at day 0. 5-FU (50 mg/kg/day) was injected intravenously at 2-day intervals, starting from day 1 to day 13. Group A received physiological saline (the vehicle of 5-FU). At days 14, 16, and 19 mice were sacrificed and the blood serum was collected. Tongue and jejunum were retrieved at necropsy. Abbreviations: BS, post-mortem blood sampling; Bio, post-mortem biopsy; OMI, oral mucositis index scoring.


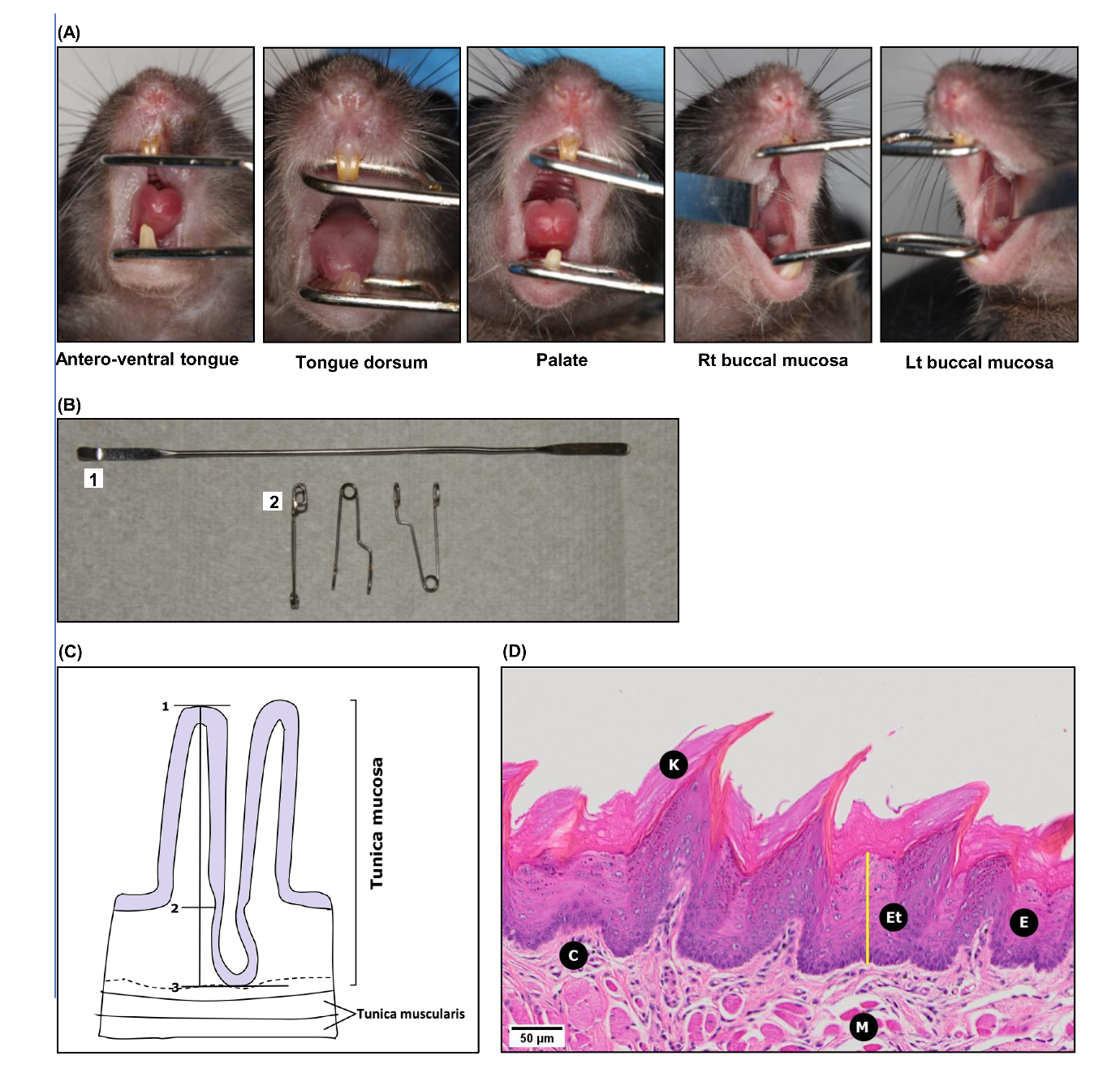


**Figure S5. Assessment of Oral Mucositis*.* A)** Oral cavity examination views. Abbreviations: Rt, Right; Lt, Left. **B)** Oral cavity examination tools; 1: Stainless steel laboratory micro spatula; 2: three views of Celentano Murine Mouth Opener (CMMO). **C)** Diagram of the wall of the small intestine demonstrating the morphometric measurements of the tunica mucosa, intestinal villi and crypts of the ileum. 1-3: Thickness of the tunica mucosa; 1-2: Length of the intestinal villi; 2-3: Depth of the intestinal crypt. **D)** Representative photomicrographs (X 20) of dorsal tongue mucosa with H&E stain, showing keratin layer (K), epithelial tissue (E), connective tissue (C) and muscle tissue (M). Epithelial thickness (Et) was measured from basal membrane to epithelial granular layer (scale bar represents 50 μm).

**Supplementary tables**

**Table S1. Difference in cell viability (% of control) of OKF6 cells treated with HA constituents compared to H_2_O_2_-treated group.** OKF6 cells were pre-treated with HA constituents (HA) for 24 hours (Mucosamin^®^ 1% v/v, H-MW-HA 0.01% w/v, Xl-HA 5/5 0.01% v/v, Xl-HA 30/30 0.01% v/v, and Xl-HA 100/100 0.01% v/v), and then were co-incubated with the same HA constituents with or without H_2_O_2_ (400 µM) for further 24 hours. At the end of experiment, cell viability was quantified by fluorescein diacetate (FDA) fluorescent assay and expressed as the relative response (%) of treated cells as compared to untreated controls ( as control cell viability = 100%). Data are expressed as means ± SD of readings from three wells per data point (technical triplicate). * p < 0.05 compared to H_2_O_2_-treated group.

| HA constituents | Cell viability (% of control) | Difference in cell viability (% of control) compared to H_2_O_2_ (400 μM) treated group |
| --- | --- | --- |
| H-MW-HA | 81.627 ± 2.491 | 22.7 ± 6.26⁎ |
| Xl-HA 30/30 | 80.775 ± 6.142 | 21.8 ± 10.6⁎ |
| Xl-HA 100/100 | 78.632 ± 10.499 | 19.7 ± 7.01⁎ |
| Mucosamin^®^ | 76.661 ± 8.656 | 17.7 ± 8.25⁎ |
| Xl-HA 5/5 | 73.324 ± 2.853 | 14.4 ± 4.16⁎ |

**Table S2. Protective effect (in ratio) of H-MW-HA 0.01% w/v and 0.05% w/v against H_2_O_2_-induced ROS production.** Data are from 3 independent experiments (three biological replicates), n= 4 per group (four technical replicates). Values represent mean (folds increase) ± SD.

| **Time plateau - 1 (0.5 hrs - 1 hrs)** | | | | | |
| --- | --- | --- | --- | --- | --- |
| **Pairs** | **Groups** | **Mean** | **SD** | **P-value** | **Protection (%)** |
|  | H_2_O_2_ (400 μM) | 5.3292 | 1.1347 |  |  |
|  | H-MW-HA 0.01% (w/v) + H_2_O_2_ (400 μM) | 4.9654 | 0.8895 |  |  |
|  | H-MW-HA 0.05% (w/v) + H_2_O_2_ (400 μM) | 4.1092 | 1.2685 |  |  |
| **Pair 1** | [H_2_O_2_ (400 μM)] - [H-MW-HA 0.01% (w/v) + H_2_O_2_ (400 μM)] | 0.3638 | 0.5148 | p < 0.001 | 6.83 |
| **Pair 2** | [H_2_O_2_ (400 μM)] - [H-MW-HA 0.05% (w/v) + H_2_O_2_ (400 μM)] | 1.2202 | 0.6334 | p < 0.001 | 22.9 |
| **Time plateau - 2 (3 hrs - 4.5 hrs)** | | | | | |
| **Pairs** | **Groups** | **Mean** | **SD** | **P-value** | **Protection (%)** |
|  | H_2_O_2_ (400 μM) | 9.2355 | 1.7589 |  |  |
|  | H-MW-HA 0.01% (w/v) + H_2_O_2_ (400 μM) | 8.1737 | 1.1996 |  |  |
|  | H-MW-HA 0.05% (w/v) + H_2_O_2_ (400 μM) | 7.0498 | 1.9351 |  |  |
| **Pair 1** | [H_2_O_2_ (400 μM)] - [H-MW-HA 0.01% (w/v) + H_2_O_2_ (400 μM)] | 1.0618 | 0.9263 | p < 0.001 | 11.5 |
| **Pair 2** | [H_2_O_2_ (400 μM)] - [H-MW-HA 0.05% (w/v) + H_2_O_2_ (400 μM)] | 2.1856 | 1.1394 | p < 0.001 | 23.67 |

**Table S3. Treatment allocations and body weights from HA Treated Mice.** Mice were treated with high molecular weight hyaluronic acid (H-MW-HA) 0.01% w/v in drinking water a day before starting 5-FU injections and the administration persistent throughout the remainder of the study. Body weight was measured every 24-48 hrs. N: number. * p <.05 vs 5-FU; ** p <0.05 vs 5-FU+H-MW-HA.

| **Days** | **Groups** | **N** | **Mean ± SD** | **Days** | **Groups** | **N** | **Mean ± SD** |
| --- | --- | --- | --- | --- | --- | --- | --- |
| **Body weight (gram) at Day-0** | **Control** | **12** | **17.5±1.32** | **Body weight (gram) at Day-13** | **Control** | **12** | **17.97±1.18** *,** |
|  | **H-MW-HA** | **12** | **17.35±1.32** |  | **H-MW-HA** | **12** | **18.65±1.17** *,** |
|  | **5-FU** | **18** | **17.4±0.78** |  | **5-FU** | **15** | **15.85±1.09** |
|  | **5-FU + H-MW-HA** | **18** | **17.64+1.28** |  | **5-FU + H-MW-HA** | **16** | **15.92±1.55** |
|  | **Total** | **60** | **17.48+1.14** |  | **Total** | **55** | **16.94±1.75** |
| **Body weight (gram) at Day-2** | **Control** | **12** | **17.86±1.19** | **Body weight (gram) at Day-14** | **Control** | **12** | **18.05±1.23***,** |
|  | **H-MW-HA** | **12** | **17.81±1.07** |  | **H-MW-HA** | **12** | **8.43±1.11** *,** |
|  | **5-FU** | **18** | **17.52±0.86** |  | **5-FU** | **15** | **15.12±1.30** |
|  | **5-FU + H-MW-HA** | **18** | **17.75±1.26** |  | **5-FU + H-MW-HA** | **16** | **15.16±1.82** |
|  | **Total** | **60** | **17.72±1.08** |  | **Total** | **55** | **16.49±2.08** |
| **Body weight (gram) at Day-4** | **Control** | **12** | **17.64±1.27** | **Body weight (gram) at Day-15** | **Control** | **8** | **17.9±1.49** *,** |
|  | **H-MW-HA** | **12** | **18.01±1.04** |  | **H-MW-HA** | **8** | **18.55±1.23** *,** |
|  | **5-FU** | **18** | **17.23±0.86** |  | **5-FU** | **10** | **14.44±0.99** |
|  | **5-FU + H-MW-HA** | **18** | **17.43±1.22** |  | **5-FU + H-MW-HA** | **8** | **15.83±1.48** |
|  | **Total** | **60** | **17.53±1.11** |  | **Total** | **34** | **16.55±2.10** |
| **Body weight (gram) at Day-6** | **Control** | **12** | **17.77±1.28** | **Body weight (gram) at Day-16** | **Control** | **8** | **17.98±1.40** *,** |
|  | **H-MW-HA** | **12** | **18.1±1.16** * |  | **H-MW-HA** | **8** | **18.91±1.20** *,** |
|  | **5-FU** | **18** | **16.82±0.83** |  | **5-FU** | **9** | **14.02±1.41** |
|  | **5-FU + H-MW-HA** | **18** | **17.08±1.17** |  | **5-FU + H-MW-HA** | **8** | **14.68±2.04** |
|  | **Total** | **60** | **17.35±1.18** |  | **Total** | **33** | **16.33±2.58** |
| **Body weight (gram) at Day-8** | **Control** | **12** | **17.48±1.18** | **Body weight (gram) at Day-17** | **Control** | **4** | **17.72±0.73** * |
|  | **H-MW-HA** | **12** | **18.15±1.12** * |  | **H-MW-HA** | **4** | **18.37±1.04** * |
|  | **5-FU** | **18** | **16.78±0.77** |  | **5-FU** | **5** | **14.5±1.70** |
|  | **5-FU + H-MW-HA** | **18** | **17.18±1.02** |  | **5-FU + H-MW-HA** | **3** | **16.83±1.62** |
|  | **Total** | **60** | **17.32±1.10** |  | **Total** | **16** | **16.71±2.02** |
| **Body weight (gram) at Day-10** | **Control** | **12** | **17.68±1.19** * | **Body weight (gram) at Day-18** | **5-FU** | **4** | **14.72±1.97** |
|  | **H-MW-HA** | **12** | **18.24±1.20** *,** |  | **5-FU + H-MW-HA** | **3** | **16.86±2.00** |
|  | **5-FU** | **18** | **16.25±1.27** |  | **Total** | **7** | **15.64±2.14** |
|  | **5-FU + H-MW-HA** | **18** | **16.72±1.34** | **Body weight (gram) at Day-19** | **Control** | **4** | **18.15±0.88** |
|  | **Total** | **60** | **17.07±1.45** |  | **H-MW-HA** | **4** | **19.05±1.02** * |
| **Body weight (gram) at Day-11** | **5-FU** | **16** | **16.81±1.12** |  | **5-FU** | **4** | **14.5±2.58** |
|  | **5-FU + H-MW-HA** | **17** | **16.85±1.28** |  | **5-FU + H-MW-HA** | **3** | **16.9±2.86** |
|  | **Total** | **33** | **16.83±1.19** |  | **Total** | **15** | **17.16±2.51** |
| **Body weight (gram) at Day-12** | **Control** | **12** | **18.01±1.35** *,** |  | | | |
|  | **H-MW-HA** | **12** | **18.57±1.16** *,** |  |  |  |  |
|  | **5-FU** | **16** | **16.46±1.21** |  |  |  |  |
|  | **5-FU + H-MW-HA** | **16** | **16.51±1.19** |  |  |  |  |
|  | **Total** | **56** | **17.26±1.50** |  |  |  |  |

**Table S4. Description of diarrheal status by treatment groups.** Mice received a single intravenous (IV) 5-FU injection (50 mg/kg/day), every 48 hours, from day 1 to day 13, plus hyaluronic acid (H-MW-HA) compound (0.01% w/v) orally in drinking water from day 0 until the endpoint day. Diarrhea was assessed every 24-48 hrs. n=60.

|  | | | **Diarrhea status** | | | | **Total** | |
| --- | --- | --- | --- | --- | --- | --- | --- | --- |
|  |  |  | **Absent** | | **Present** | |  |  |
|  |  |  | **Count** | **% within treatments groups** | **Count** | **% within treatments groups** | **Count** | **% within treatments groups** |
| **Treatments groups** | **Control** | | 12 | 100% | 0 | 0.0% | 12 | 100% |
|  | **H-MW-HA** | | 12 | 100% | 0 | 0.0% | 12 | 100% |
|  | **5-FU** | | 5 | 27.8% | 13 | 72.2% | 18 | 100% |
|  | **5-FU+H-MW-HA** | | 17 | 94.4% | 1 | 5.6% | 18 | 100% |
|  | | **Total** | 46 | 76.7% | 14 | 23.3% | 60 | 100% |

**Table S5.** **The effect of H-MW-HA on the 5-FU-induced intestinal injury.** Descriptive statistics of morphometric variables of the jejunum wall of C57BL/6 mice following chemotherapy and Hyaluronic acid (HA) treatment. Groups were treated with saline via tail vein (IV) (control), H-MW-HA (0.01%) in the drinking water (H-MW-HA), 5-FU (50 mg/kg) IV (5-FU), or H-MW-HA/5-FU (5-FU+H-MW-HA). Data presented are means ± SD of 12 representative measurement/tissue (three representative mice/group). (a) denoted p < 0.05 compared to control and H-MW-HA groups. (b) denoted p < 0.05 compared to the 5-FU group. (c) denoted p < 0.05 compared to the control group.

| **Treatment groups** | | **Morphometric measurement (µm)**  **Tunica mucosa thickness Villi length Crypts depth** | | |
| --- | --- | --- | --- | --- |
| **Control** | |  |  |  |
| **Day-14** | 602.43±24.19 | 507.20±21.72 | 95.09±6.76 |  |
| **Day-16** | 611.92±42.08 | 508.74±48.67 | 114.09±6.25 |  |
| **Day-19** | 631.06±81.28 | 519.83±83.13 | 118.37±22.25 |  |
| **Day14-19** | 615.14±48.98 | 511.92± 49.73 | 109.18±16.13 |  |
| **H-MW-HA** | |  |  |  |
| **Day-14** | 590.4±37.41 | 507.7±26.37 | 87.95±6.96 |  |
| **Day-16** | 620.34±31.93 | 509.6±33.28 | 108.85±1.74 |  |
| **Day-19** | 574.02±20.26 | 484.7±17.51 | 92.87±4.52 |  |
| **Day14-19** | 594.92±33.48 | 500.66±25.93 | 96.56±10.37 |  |
| **5-FU** | |  |  |  |
| **Day-14** | 390.9±19.99**^a^** | 298.8±13.75**^a^** | 89.59±13.45 |  |
| **Day-16** | 405.86±25.04**^a^** | 308.7±4.98**^a^** | 91.60±11.78**^c^** |  |
| **Day-19** | 424.71±7.09**^a^** | 323.5±10.51**^a^** | 95.88±11.67 |  |
| **Dya14-19** | 406.86±22.26**^a^** | 310.35±14.02**^a^** | 92.36±11.03**^c^** |  |
| **5-FU+H-MW-HA** | |  |  |  |
| **Day-14** | 472.28± 21.61**^b;c^** | 377.23±20.40**^a;b^** | 98.38±8.97 |  |
| **Day-16** | 523.94±21.59**^b;c^** | 418.23±27.53**^a;b^** | 105.16±8.90 |  |
| **Day-19** | 599.61±37.09**^b^** | 507.87±36.97**^b^** | 99.40±5.76 |  |
| **Day14-19** | | 531.94±60.44**^b;c^** | 434.44±63.11**^a;b^** | 100.98±7.63 |

**Table S6. Visual oral ulcerative mucositis score (1).** Based on a modification of the method of Sonis et al. (2).

| Grade | Severity | Description |
| --- | --- | --- |
| **0** |  | Normal (no abnormalities) |
| **1** | Mild | Partial hyperemia, erythema and swelling, but no evidence of mucosal erosion (mucosa intact). |
| **2** | Mild | Overall hyperemia, erythema and swelling and superficial erosion, such that there may be mild sloughing. |
| **3** | Moderate | Epidermolysis, hyperemia and erythema; moderate mucositis characterized by frank ulcer formation. Ulcers typically have areas of necrosis (erosion) with associated yellowish/grey coloration with pseudomembrane formation. Cumulative area of ulceration ≤ 25% of tongue surface area. |
| **4** | Severe | Extensive epidermolysis and bleeding; severe mucositis with ulcer formation affecting 25% to 50% of tongue or buccal mucosal mucosal area. Marked erythema and pseudomembrane formation. Loss of pliability. |
| **5** | Severe | Bleeding and abscesses; virtually complete ulceration of the mucosa, all mucosa areas at risk. Loss of mobility and pliability. |

1. Nakajima N, Watanabe S, Kiyoi T, Tanaka A, Suemaru K, Araki H. Evaluation of edaravone against radiation-induced oral mucositis in mice. J Pharmacol Sci. 2015;127(3):339-43.

2. Sonis ST, Peterson RL, Edwards LJ, Lucey CA, Wang L, Mason L, et al. Defining mechanisms of action of interleukin-11 on the progression of radiation-induced oral mucositis in hamsters. Oral Oncology. 2000;36(4):373-81.
